# Soy Protein-Based Hydrogels: Recent Advances in Molecular Design and Functional Applications

**DOI:** 10.3390/gels12090761

**Published:** 2026-08-25

**Authors:** Zhongjian Li, Luohui Wang, Liyun Wang, Man Yin, Lin Zhang, Xian Wang, Limin Guo, Xiangmeng Chen, Cheng Li

**Affiliations:** 1 College of Horticulture, College of Forestry, College of Chemistry and Materials, Henan Agricultural University, Zhengzhou 450026, Chinawin5823@163.com (L.W.);; 2 College of Pharmacy, Changsha Medical University, Changsha 410219, China

**Keywords:** soy protein hydrogels, 7S/11S globulin, sustainable materials, molecular design, crosslinking strategies, structure–property relationship, functional applications

## Abstract

To address the limitations of conventional polymer hydrogels in terms of sustainability and functionality, green soy protein (SP)-based hydrogels (SPHs) demonstrate significant potential. As an abundant, renewable plant protein, SP provides an ideal molecular platform for constructing high-performance, multifunctional hydrogels. This review systematically consolidates recent progress in SPHs. Firstly, the molecular fundamentals and gelation mechanisms of soy protein are analyzed in depth. Subsequently, key construction strategies, including physical, chemical, and enzymatic crosslinking, as well as composite/hybrid approaches, are comprehensively reviewed with respect to their mechanisms, advantages, and limitations. Following this, innovative applications of SPHs in biomedical, food and nutrition, environmental/agricultural, and smart material fields are highlighted. Finally, the current challenges facing research in mechanical properties, structure–property relationships, and scalable production are identified. Future directions include developing novel green crosslinking systems, deepening multi-scale structural control, and advancing smart integrated design. This review aims to provide researchers with a systematic knowledge framework spanning from “molecular understanding” to “functional customization,” thereby propelling soy protein hydrogels toward higher toughness, intelligence, and sustainability.

## 1. Introduction

The depletion of fossil resources and the escalating environmental pollution necessitate the development of sustainable, environmentally friendly bio-based materials as a critical direction in materials science. In the wave of green transformation toward next-generation materials, natural polymer hydrogels have demonstrated immense potential for applications in frontier areas such as biomedicine, flexible electronics, food engineering, and environmental remediation, owing to their high water content, extracellular matrix-like structure, and excellent biocompatibility [1,2,3]. However, traditional natural polymer hydrogels are often constrained by raw material costs, supply stability, and batch-to-batch variation, while synthetic polymer hydrogels, due to their reliance on petrochemical resources and potential non-biodegradability, struggle to meet the fundamental requirements of a circular economy and sustainable development [4,5,6]. Consequently, identifying a natural biopolymer that is widely sourced, renewable, cost-effective, and possesses outstanding functionality is pivotal for advancing next-generation functional hydrogels.

Soy protein (SP) is a highly promising raw material for developing a new generation of environmentally friendly, functionally active hydrogels. As a major by-product of the global soybean processing industry, SP is exceptionally abundant and low-cost, offering significant advantages in terms of renewability and carbon footprint [7,8]. More importantly, its unique molecular structure provides a natural platform for sophisticated molecular design. SP is primarily composed of two major fractions: β-conglycinin (7S) and glycinin (11S). Their polypeptide chains are densely packed with abundant functional groups, including amino, carboxyl, thiol, and hydroxyl groups. These moieties not only serve as the cornerstone for forming three-dimensional networks *via* physical/chemical crosslinking but also provide numerous reactive sites for introducing new functionalities through chemical modification [9,10,11].

Furthermore, SP inherently contains bioactive sequences, such as arginine–glycine–aspartic acid (RGD), which can promote cell adhesion and proliferation, granting it unique advantages over many other plant proteins in biomedical applications, such as tissue engineering [12,13]. Compared with extensively studied animal-derived proteins, SP avoids potential religious taboos and the risk of pathogen transmission, offering superior public acceptance and biosafety. Compared with prolamin proteins, it exhibits greater water solubility and an amphiphilic structure, which are more conducive to mild gelation and processing in aqueous phases. Translating these inherent molecular advantages into three-dimensional network-structured soy protein hydrogels (SPHs) through a controlled sol–gel transformation enables SP to leap from a raw material to a high-value-added functional material. SPHs not only inherit the inherent bioactivity and biodegradability of SP but also, through precise molecular design, acquire tunable mechanical properties, porous structures, and intelligent responsiveness to environmental stimuli such as pH, temperature, and ionic strength. These characteristics enable them to transcend the application boundaries of traditional materials, offering broad prospects for use as drug delivery carriers, tissue-engineering scaffolds, smart food packaging, and environmental sorbents [14,15,16,17].

Despite the immense application potential of soy protein hydrogels, key challenges remain in translating SPHs from fundamental design to practical application. Firstly, the gelation process of SP involves complex dynamics of protein denaturation, aggregation, and network formation. The structure–property relationships between molecular conformation and the macroscopic mechanical properties, swelling behavior, and stability of the final hydrogel remain incompletely elucidated. Such an incomplete understanding often results in pure SP hydrogels exhibiting brittleness, low mechanical strength, and susceptibility to syneresis, severely limiting their practical application [18]. Secondly, to achieve specific functionalities, researchers have explored a wide range of strategies, including physical, chemical, and enzymatic crosslinking, nanocomposite reinforcement, and double-network construction. However, these studies are often scattered across different application branches, lacking a systematic scientific synthesis [19,20,21]. Notably, how to effectively and synergistically enhance the mechanical strength, toughness, and water-holding capacity of SP without compromising its inherent biocompatibility through structural engineering remains a central challenge.

In this context, the present review aims to systematically integrate and critically assess recent breakthroughs of SPHs, address the aforementioned knowledge gaps, and provide a clear overview of this interdisciplinary research frontier. This article first investigates the molecular structural characteristics of SP and its gelation mechanisms. Subsequently, it comprehensively categorizes and elaborates on the mechanisms and limitations of physical, chemical, and biological crosslinking strategies, with a focus on advanced methods for functional modification through composite formation with polysaccharides, synthetic polymers, and nanomaterials. On this basis, the article systematically summarizes innovative applications of SPHs in biomedicine, food science, environmental remediation, and emerging fields. We expect that this work not only provides a comprehensive knowledge framework from “molecular design” to “functional realization,” but also inspires new research ideas. It is hoped that these efforts will collectively advance soy protein hydrogels from the laboratory to broader practical applications, contributing material science solutions to global sustainable development goals.

In this context, it is important to distinguish our work from several recently published reviews on soy protein-based materials. Other reviews often focus on a single application, such as drug delivery or tissue engineering, and therefore lack the comprehensive “molecular design to functional application” perspective we aim to provide. Our review is novel in three key aspects: (1) it presents a unified comparative framework that critically assesses physical, chemical, enzymatic, and composite/hybrid strategies using qualitative performance criteria, providing researchers with practical guidance for strategy selection; (2) it systematically surveys emerging frontiers, including flexible sensors, energy storage devices, and bio-inspired adhesives, which have not been comprehensively reviewed in the context of soy protein hydrogels; and (3) it identifies key translational gaps such as raw material variability, immunogenicity, sterilization, and regulatory approval, while proposing a forward-looking, multi-tiered roadmap for industrial translation. By bridging the gap between molecular engineering and functional applications across multiple sectors, this review aims to serve as a definitive reference that not only consolidates current knowledge but also inspires future innovations.

## 2. Molecular Fundamentals of Soy Protein

The formation of soy protein hydrogels is essentially a phase transition in which dispersed soy protein molecules undergo conformational unfolding and intermolecular interactions under specific conditions, ultimately forming a three-dimensional network that spans the entire system. The realization and precise control of this process are rooted in the unique molecular composition of soy protein and its responsiveness to external environments. This chapter investigates the molecular structure of soy protein and its key functional groups, laying the foundation for a systematic discussion of the physicochemical mechanisms of gel formation and molecular design strategies (Table 1).

### 2.1. Molecular Structural Characteristics of Soy Protein

Soy protein is a complex, multicomponent mixture typically extracted from defatted soybean meal using an alkali solubilization and acid precipitation process (Table 1). Based on their ultracentrifugation sedimentation coefficients, they are classified as β-conjugated soy globulin (7S) and glycine (11S), which together account for 70–80% of the total protein content and are the key components that determine gelation behavior [22]. In addition, SPI contains small amounts of other protein components and non-protein substances, which may affect protein solubility and gel behavior. By adjusting the pH and ionic strength of the extraction solution and employing techniques such as isoelectric precipitation, membrane separation, or chromatography, it is possible to further enrich or isolate high-purity 7S or 11S fractions to study their unique gel properties [23]. Table 1 provides a systematic summary of the major components, abundance, extraction methods, and biological significance of soybean protein. The 7S globulin is a trimeric glycoprotein with a molecular weight of approximately 150–200 kDa, assembled from α, α’, and β subunits through non-covalent interactions, with an isoelectric point around pH 4.8 [24]. Its structure lacks disulfide bonds, and conformational stability relies on hydrogen bonds and hydrophobic interactions. It has a relatively low thermal denaturation temperature (approximately 65–75 °C). Upon heating, it unfolds more readily, exposing hydrophobic and active thiol groups and tending to form chain-like and branched aggregates. Such aggregation behavior confers good water-holding capacity and transparency to the gel but often results in reduced mechanical strength [25].

**Table 1:** Main Components of Soy Protein: Abundance, Extraction Methods, and Biological Significance.

	Abbreviation/Symbol	Approximate Abundance (% of Total Protein)	Molecular Characteristics	Extraction/Purification Methods	Biological Significance	Refs
β-conglycinin	7S	30–40%	Trimeric glycoprotein, 150–200 kDa; no disulfide bonds; pI ~4.8; Td 65–75 °C	Dissolved in a low ionic strength (μ = 0.5) Tris-HCl buffer (pH 7.5), separated by centrifugation	Contributes to flexibility and water-holding capacity during gel formation; contains major allergenic epitopes; relatively low thermal stability	[22,24,25]
Glycinin	11S	30–40%	Hexamer, 300–380 kDa; contains interchain disulfide bonds; pI 4.5–5.0; Td 85–95 °C	Precipitated under low ionic strength conditions, collected by centrifugation	Contributes to strength and rigidity during gel formation; contains major allergenic epitopes; relatively high thermal stability	[22,26,27]
2S globulin	2S	8–15%	Small molecular weight protein (~20 kDa); rich in sulfur-containing amino acids	Salting out followed by ultrafiltration or chromatographic separation	Nutritional supplementation; contains minor allergenic epitopes	[22,28]
Lipoxygenase	LOX	1–2%	Molecular weight ~94–102 kDa; contains non-heme iron	Isoelectric precipitation or ion exchange chromatography	Catalyzes the oxidation of polyunsaturated fatty acids; produces beany flavor volatiles	[28,29]
Trypsin inhibitors	KTI/BBI	5–10%	Kunitz (~21 kDa) and Bowman–Birk (~8 kDa) inhibitors	Affinity chromatography or thermal inactivation (heat treatment)	Anti-nutritional factors; require heat treatment for inactivation; potential anticancer activity	[29]
Lectins	Soybean agglutinin	~1%	Tetramer, ~120 kDa; binds N-acetylgalactosamine	Affinity chromatography	Anti-nutritional factors; require heat treatment for inactivation	[29]

In contrast, the 11S globulin is a hexamer with a molecular weight of approximately 300–380 kDa. It consists of acidic (A) and basic (B) subunits paired *via* interchain disulfide bonds, assembled into two ring-like structures, with an isoelectric point around pH 4.5–5.0 [26,27]. Its structure is highly compact, with a stable hydrophobic core, and free thiol groups are present on the molecular surface. Upon heating or under oxidative conditions, new intermolecular disulfide bonds form *via* thiol–disulfide exchange, which is the key chemical basis for gels achieving high mechanical strength. The 11S fraction has a high thermal denaturation temperature (85–95 °C). Upon thermal unfolding, it rapidly forms tight particulate aggregates that contribute to network rigidity and thermal stability, but potentially at the expense of water-holding capacity and transparency. Evidently, 7S and 11S play complementary roles in gel construction—the former contributes flexibility and water-holding capacity, while the latter provides strength and rigidity. Therefore, regulating the 7S/11S ratio in the raw material or performing specific component blending enables refined design of gel network structure and properties, a strategy we term “component engineering.” This concept highlights that the macroscopic properties of the final hydrogel are not merely a sum of the properties of its individual components but are critically dependent on their relative abundance and interactions. Simultaneously, environmental factors, such as solution pH and ionic strength, influence molecular aggregation pathways and network homogeneity by modulating the balance between electrostatic repulsion and hydrophobic interactions.

Although soy protein contains a variety of components, this review focuses on the 7S and 11S components. First, this is because they are the primary functional components constituting the SPI gel network; their content ratios, molecular structures, and aggregation behavior directly determine the macroscopic properties of the final hydrogel. Second, the structural and functional characteristics of 7S—which contribute flexibility and water-holding capacity—complement those of 11S—which provides strength and rigidity—making them the most versatile structural units in hydrogel construction [28]. In contrast, other trace protein components make a smaller contribution to the gel network, playing either a minor role (such as lipoxygenase, which acts as a flavor contributor rather than a gel-forming agent) [29]. Therefore, understanding the unique characteristics of these two components is crucial for the rational design of soy protein-based hydrogels with tailored properties.

### 2.2. Active Functional Groups in Soy Protein

The wealth of active functional groups in soy protein endows it with immense potential for physical, chemical, and covalent crosslinking, providing the chemical basis for the precise construction and functional customization of gel networks. Table 2 systematically summarizes the five main functional groups in soy protein and their roles in gel crosslinking.

## 3. Construction Strategies for Hydrogels

The unique molecular structure of soy protein confers inherent advantages for forming hydrogels through various mechanisms. Based on the nature of the crosslinking bonds, construction strategies can be categorized into four major classes: physical, chemical, enzymatic, and composite/hybrid. Each category corresponds to distinct gel characteristics and application scenarios. This chapter will systematically review and critique the intrinsic mechanisms, recent advances, and roles of these key construction strategies in shaping gel properties.

Before reviewing specific composite systems, it is important to note a critical methodological challenge: the mechanical performance data (e.g., tensile strain, compressive strength, modulus) reported in the literature are often obtained under vastly different experimental conditions, including protein concentration, crosslinker dosage, testing instrument types, strain rates, and environmental conditions. Consequently, any direct quantitative comparison of absolute values across different studies must be made with caution. The following discussion therefore focuses on qualitative trends and mechanistic insights rather than direct comparisons of raw data, unless explicitly noted otherwise.

### 3.1. Physical Crosslinking Methods

Physical crosslinking is a core strategy for constructing soy protein hydrogels. Its essence lies in harnessing the synergistic effects of non-covalent interactions to assemble protein molecules into a three-dimensional network under mild conditions. Compared with chemical crosslinking, these methods can best preserve the native conformation and bioactivity of soy protein. However, networks that rely solely on non-covalent physical crosslinking often exhibit insufficient mechanical strength and environmental stability, constituting a long-standing core paradox in this field. This reflects the broader trade-off between crosslinking density and functional performance, which is discussed in detail in Section 3.6. To address this bottleneck, researchers have systematically explored multiple dimensions, including regulating intermolecular interaction forces, introducing synergistic components, and developing novel physical processing technologies.

Early physical gelation strategies primarily relied on heat-induced aggregation and ionic crosslinking. Heat-induced aggregation involves heating to denature the protein, expose hydrophobic groups, and form intermolecular disulfide bonds, with the network stabilized upon cooling by hydrogen bonds and hydrophobic interactions. However, the microscopic structure of such gels is often difficult to control. Ionic crosslinking uses polyvalent cations such as Ca^2+^ and Zn^2+^ to form “ionic bridges” with carboxyl groups on protein chains, enabling rapid network formation with relatively high hardness and water-holding capacity. However, this process is highly sensitive to ion concentration, and excessive divalent ions can easily trigger over-aggregation, leading to coarse, opaque, non-ideal gel structures. Li et al. [33], in their study on the gelation of deamidated soy protein, explicitly revealed this phenomenon: the order of ion valence effectiveness in enhancing gel strength was Ca^2+^ > Mg^2+^ > Na^+^.> Ca^2+^, while enhancing strength, also most readily causes phase separation and heterogeneity. To overcome these limitations, introducing polysaccharides to construct protein–polysaccharide electrostatic complex hydrogels represents a significant paradigm shift. This strategy forms dense, stable composite networks *via* electrostatic attraction, thereby improving mechanical strength and stability. Wang et al. [37] ingeniously introduced the Hofmeister effect into an SPI–κ-carrageenan hybrid system. They found that kosmotropic-type SO_4_^2−^ significantly enhanced the gel network by strengthening hydrophobic hydration, while chaotropic-type SCN^−^ had a destructuring effect. And they optimized a key parameter: the salt-to-protein concentration ratio to 2:1.

In the realm of physical-field-assisted technologies, the emergence of high-energy ultrasound and electric-field processing marks a shift in gel construction strategies from passive reliance on molecular self-assembly to active directional manipulation. Ultrasonication treatment disrupts protein aggregates instantaneously *via* cavitation and shear forces, exposing hydrophobic and thiol groups and thereby “activating” reactive sites. Cheng et al. [38] pioneered the unique advantages of short-time, high-intensity ultrasonication: only 5 min of treatment, combined with 1% glucono-δ-lactone, could directly induce gelation without additional temperature control. The gel network exhibited more β-sheet structure, enhanced hydrophobic interactions, and disulfide bonds. Similarly, Chen et al. [39] combined ultrasonication with κ-carrageenan and NaCl. They found that 525 W ultrasonication for 15 min dramatically increased hydrophobic group exposure by 161.1%, precisely constructing a composite network with high hardness and elasticity through synergistic enhancement of multiple non-covalent interactions. Electric field processing demonstrates the ability to construct anisotropic structures. Zhu et al. [40] induced the formation of anisotropic hydrogels with oriented structures from soy protein and konjac glucomannan by applying a low-voltage electric field; Wang et al. [41] further developed an alternating electric field (AEF) pretreatment strategy, utilizing AEF to induce protein conformation unfolding and release thiol groups, subsequently compounding with rice bran wax oleogel to prepare a high-performance bigel system. High-pressure processing also offers a unique dimension for control. Florowska et al. [42] found that high hydrostatic pressure (HHP) had a nonlinear impact on inulin–soy protein composite gels: 300 MPa strengthened non-covalent interactions, making the network denser, whereas 500 MPa destabilized the network. These findings identified 300 MPa for 5 min as the optimal processing condition.

Recently, employing purely physical pH-shifting and co-folding strategies to achieve precise molecular design of higher-order protein structures has emerged as a more promising research direction. Inspired by protein folding principles, Guo et al. [43] completely unfolded soy protein and shellac under alkaline conditions, then adjusted the pH back to neutral to induce co-folding of the two macromolecules, forming a porous gel network governed entirely by disulfide bonds and hydrophobic interactions. The breakthrough of this method lies in elevating the control of the gelation process to the level of conformational transitions in protein secondary and even tertiary structures. Ji et al. [35] found that sericin, as a flexible co-gelling agent, at an optimal addition level of 15% could promote soy protein unfolding and enhance thiol–disulfide exchange, forming a more uniform and dense network and increasing water-holding capacity by approximately 25%. However, excessive addition led to excessive hydrophobic aggregation and steric hindrance, disrupting network homogeneity. Therefore, the effectiveness of synergistic strategies depends heavily on the precise regulation of component ratios.

In summary, the advantage of physical cross-linking strategies is that they involve mild processing conditions and preserve protein bioactivity; however, hydrogels relying solely on noncovalent interactions typically exhibit insufficient mechanical strength and environmental stability. The introduction of polysaccharides and physical field-assisted techniques has expanded the design space, but the fundamental trade-off between mild processing and mechanical performance remains unresolved. Among physical methods, heat-induced gelation remains the most practical approach for large-scale applications due to its simplicity, whereas ultrasound-assisted and pH-shifting methods offer better control over the network structure but increase processing complexity and energy consumption.

### 3.2. Chemical Crosslinking Methods

Chemical crosslinking involves introducing covalent bonds to permanently link soy protein molecular chains, constituting a fundamental approach for constructing high-strength, high-stability hydrogels. Compared to physical crosslinking, covalent bonds impart excellent mechanical rigidity, anti-swelling properties, and resistance to degradation in gels. However, this performance advantage comes with concerns about introducing exogenous reagents that may affect biocompatibility and the risk of losing the native protein conformation under harsh reaction conditions. Therefore, balancing “efficient crosslinking” with “biological friendliness” and integrating new functionalities while enhancing mechanical properties remain central themes in this field.

Traditional chemical crosslinking primarily relied on small-molecular aldehydes, such as glutaraldehyde, achieving efficient crosslinking *via* Schiff base condensation between aldehyde groups and the ε-amino groups of lysine residues. However, their cytotoxicity severely limits their applications in the food and biomedical fields. In recent years, the research trend has shifted from selecting low-toxicity crosslinkers to pursuing the overall greenness and controllability of the reaction system. Oxidized polysaccharide macromolecular crosslinking platforms are a prominent example. Cheng et al. [30] pioneered the use of oxidized sodium alginate as a macromolecular crosslinker, thereby forming a three-dimensional network via dynamic Schiff base bonds without heating or additional additives. They revealed that tuning the degree of oxidation of the polysaccharide could precisely control the key structure–property relationship in the network’s microscopic topology. Xu et al. [31] and Liu et al. [32] employed oxidized dextran and oxidized dextrin as multifunctional crosslinking hubs, respectively, achieving synergistic crosslinking through Schiff base bonds, hydrogen bonds, Michael addition, and π-π stacking. Wang et al. [44] used dialdehyde-modified sodium alginate to fabricate structurally controllable hydrogels, thereby preliminarily demonstrating their potential for drug delivery applications. However, such macromolecular crosslinking systems also face inherent challenges regarding mechanical performance. Liu et al. [45] grafted oxidized poly(ethylene glycol) monomethyl ether onto SPI, achieving thermo-responsive injectability, but the storage modulus was only about 300 Pa. This illustrates the trade-off between limited grafting sites on macromolecular chains and high crosslinking density.

To overcome the limitations of single-component crosslinking, constructing interpenetrating polymer networks (IPNs) or semi-interpenetrating networks (semi-IPNs) has become an effective approach to substantially improve the comprehensive performance of hydrogels. Song et al. [46] developed a one-pot strategy: first functionalizing SPI to form polymerizable macromolecular monomers, then free-radical copolymerizing with potassium acrylate to form a semi-IPN structure, enabling the gel to swell stably over a wide pH range. The described approach provides an efficient technical route for protein–synthetic polymer interpenetrating networks. Li et al. [47] constructed an IPN hydrogel of SPI and carboxymethyl chitosan, using microbial transglutaminase (mTGase) and genipin as dual crosslinkers, thereby endowing the network with excellent mechanical properties, thixotropy, and pH-responsive controlled-release characteristics.

In recent years, the goal of chemical crosslinking strategies has evolved from mere mechanical enhancement toward “crosslinking-function integration.” Polyphenols and metal–phenolic networks have become versatile tools for achieving this goal. Yan et al. [48] controllably grafted gallic acid (GA) onto SPI, simultaneously constructing a tight crosslinked network and imparting antioxidant and anti-proteolytic degradation capabilities. Revealing that the amount of grafting is a key molecular switch regulating network density. Shi et al. [49] constructed an epigallocatechin gallate (EGCG)–metal ion network, increasing the storage modulus to 2.1 times that of pure SPI gel and integrating antibacterial functionality. Ding et al. [50] used cinnamaldehyde as a functional crosslinker, forming covalent bonds with SPI *via* Schiff base and Michael addition reactions. A 3% addition increased gel hardness and water-holding capacity by 126% and 22%, respectively. Additionally, Peng et al. [51] pioneered a two-step method that combines the physical crosslinking of acidic tremella polysaccharide with Lactobacillus plantarum fermentation, resulting in a fine, tough composite network with enhanced antioxidant capacity. The proposed biomanufacturing strategy offers a new perspective on the construction of protein gels.

Introducing nanofillers into chemically crosslinked networks expands the design dimensions from the molecular scale to multi-scale structural control. Jin et al. [52] designed a sophisticated “sandwich structure”: first coating SPI onto montmorillonite nanosheet surfaces, then dispersing the coated nanosheets into an SPI matrix for glutaraldehyde crosslinking. The resulting structure achieved synergy between chemical crosslinking and nano-reinforcement, increasing the storage modulus by nearly an order of magnitude. Yang et al. [53] systematically compared the reinforcing effects of pristine and carboxylated cellulose nanocrystals (CNCs) on soy protein amyloid fibril hydrogels. They found that only 0.75% of carboxylated CNCs could comprehensively improve water-holding capacity, strength, storage modulus, and thermal stability, while also warning that excessive nanoparticle concentration leads to self-aggregation rather than interfacial enhancement.

Among all strategies, chemical cross-linking provides the highest mechanical strength and stability, as covalent bonds ensure the permanent integrity of the network. However, this performance advantage comes at a high cost: the use of cytotoxic cross-linking agents limits biomedical applications, while macromolecular cross-linking systems often suffer from low cross-linking density due to a limited number of grafting sites. Approaches such as polyphenol grafting and metal–phenolic networks represent promising avenues for combining mechanical reinforcement with additional functions (such as antioxidant and antibacterial properties). However, the scalability and long-term biocompatibility of these complex systems have yet to be fully demonstrated.

### 3.3. Enzymatic Crosslinking Methods

Enzymatic crosslinking represents a frontier practice in green biomanufacturing for soy protein hydrogels. It leverages enzymes’ high substrate specificity and mild catalytic conditions to drive covalent linkages between protein molecules in near-physiological environments, thereby circumventing the cytotoxicity risks associated with traditional chemical crosslinking. Microbial transglutaminase (mTGase or TGase), due to its unique ability to catalyze the formation of stable isopeptide bonds between lysine and glutamine residues, has become a key tool enzyme in this field. The catalyzed ε-(γ-glutamyl)lysine crosslinks are highly stable and exhibit excellent biocompatibility. However, pure enzymatic crosslinking often encounters bottlenecks, including slow reaction rates and limited control over network structure. Current research focus has shifted from the application of single enzymes to integrated strategies that deeply couple enzyme catalysis with physical pretreatment, polysaccharide synergy, and functional grafting.

The efficiency of TGase crosslinking depends heavily on the substrate protein’s conformation and concentration. Liang et al. [54] found that increasing SPI concentration from 8% to 12% significantly enhanced the density and strength of the crosslinked network. Furthermore, the 7S globulin fraction, with more accessible reactive sites, yielded more stable crosslinked gels than the 11S fraction. The introduction of gelatin-enabled TGase further catalyzes covalent hybridization between 7S and gelatin molecules, yielding hybrid networks with a higher storage modulus and superior water-holding capacity. Introducing polysaccharides into TGase crosslinking systems to construct physical–chemical dual networks is another important paradigm for substantially improving gel performance. Gan et al. [55] composited microbial exopolysaccharide Salecan with SPI, first pre-denaturing *via* heat treatment, then locking the network with TGase. They discovered that Salecan concentration was a key lever for regulating network topology, and that hydrogen bonds between the polysaccharide and SPI cooperatively contributed to the formation of the interpenetrating network.

To overcome the kinetic limitations of pure enzymatic crosslinking, researchers have begun actively introducing ions or enzymatic pretreatment to pre-program protein conformation. Wang et al. [56] combined the Hofmeister effect with TGase-catalyzed crosslinking, finding that the order of different anions enhancing gel hardness was H_2_PO_4_^−^ > CH_3_COO^−^ > SO_4_^2−^ > Citrate^3−^ > Cl^−^ > CO_3_^2−^. The salt ions achieved supramolecular synergy through a “covalent–noncovalent” dual network, enhancing hydrophobic interactions and hydrogen bonds while reducing free water content. Zhao et al. [57] adopted a “modification first, crosslinking later” strategy, using laccase to catalyze the grafting of ferulic acid onto SPI, thereby increasing the α-helix content from 11.50% to 27.39%. Subsequent TGase crosslinking increased gel hardness and water-holding capacity by 50.61% and 26.21%, respectively. After simulated digestion, the DPPH and ABTS radical-scavenging rates reached 87.65% and 84.45%, respectively, demonstrating a trinity design integrating conformational regulation, crosslinking enhancement, and antioxidant functionality.

The application of enzymatic crosslinking technology has expanded beyond traditional bulk hydrogels to encompass micro- and nanoscale particles and smart, responsive materials. Guo et al. [58] homogenized bulk SPI hydrogels crosslinked by mTGase to obtain nanoscale microgel particles. Their three-phase contact angle approached 90°, enabling their use as efficient Pickering emulsion stabilizers with at least 8 weeks of storage stability under extreme pH conditions near the isoelectric point. Li et al. [59] used TGase for precise crosslinking to construct water-triggered shape memory gels. They found that 0.4% enzyme concentration achieved the most complete crosslinking, but increasing the enzyme concentration to 1.0% led to excessive crosslinking, resulting in structural damage and performance decay. The observed effects underscore the critical importance of precise enzyme dosage in controlling the outcomes of enzymatic crosslinking.

The role of enzymes in the gelation process extends beyond catalyzing covalent bond formation; they can also act as precise “molecular sculpting knives” to reshape protein self-assembly pathways. We propose this term to encapsulate the idea that enzymes, through their specific and selective activity, can precisely “carve” or modify protein molecules at defined sites, thereby redirecting the entire supramolecular assembly pathway. Li et al. [36] used protein glutaminase to deamidate SPI, converting glutamine to glutamic acid and thereby enhancing electrostatic repulsion and inhibiting the formation of large, insoluble aggregates during thermal aggregation. The resulting hydrogels exhibited exceptionally high transparency and a fine, uniform network. Lu et al. [60] demonstrated a “hydrolysis–self-assembly” pathway, using trypsin to controllably hydrolyze SPI and expose fibrillogenic motifs, thereby promoting self-assembly into β-sheet-rich nanofibers. These were subsequently crosslinked with sodium alginate to prepare composite microspheres, achieving less than 10% release in simulated gastric fluid over 48 h and up to 88.4% in simulated intestinal fluid, perfectly realizing gastro-protection and targeted delivery.

Due to their excellent biocompatibility, enzymatically crosslinked hydrogels are particularly suitable as delivery carriers for probiotics and bioactive substances. Yan et al. [61] encapsulated *Lactobacillus paracasei* LS14 in TGase-crosslinked SPI–sugar beet pectin IPN hydrogels with an encapsulation efficiency exceeding 88.9%. However, they observed that the probiotic cells themselves, due to steric hindrance, delayed the TGase crosslinking reaction, causing slight disruption of the ordered network structure. This discovery reveals the complex coupling between “payload” and “crosslinking reaction” during the loading of live bioactive substances. Predicting and controlling this coupling effect is a key scientific question that must be addressed for achieving precise delivery.

Enzymatic cross-linking offers unparalleled specificity and biocompatibility, operates under mild conditions, and preserves protein structure and biological activity. The high stability of the isopeptide bonds catalyzed by transglutaminase makes this method particularly attractive for biomedical and food applications. However, slow reaction kinetics, high enzyme costs, and substrate dependency (7S is more reactive than 11S) pose practical limitations. Current research focuses on combining enzymatic cross-linking with physical pretreatment or polysaccharide synergism to overcome kinetic bottlenecks; however, the complexity of these multi-step processes may hinder industrial translation. The emerging application of enzymes as “molecular scalpels” to reshape protein assembly pathways represents an exciting frontier that may open up new gel structures.

### 3.4. Composite Strategies

Hydrogels constructed *via* a single crosslinking mechanism often struggle to balance mechanical strength, biocompatibility, and functionality—physical crosslinking is mild and reversible but lacks mechanical performance; chemical crosslinking offers excellent strength but may sacrifice biocompatibility; enzymatic crosslinking is limited by reaction kinetics and cost. Therefore, composite strategies that synergistically integrate multiple crosslinking mechanisms and multiple components have become the core paradigm in current soy protein hydrogel design. Their essence lies in rationally combining different intermolecular forces (covalent bonds, ionic bonds, hydrogen bonds, hydrophobic interactions) with structural units such as polysaccharides, nanofillers, and synthetic polymers to achieve synergistic enhancement across multiple scales.

Combining natural polysaccharides with soy protein is the most extensively researched direction. Qi et al. [62] systematically compared the differential regulation effects of sodium alginate (SA), κ-carrageenan (KC), and xanthan gum (XG) on SPI–potato protein composite gels. They found that SA enhanced hardness and elasticity *via* Ca^2+^ ionic bridges, achieving 99.94% water-holding capacity at 10% concentration. KC, due to charge-neutralization-triggered micro-phase separation, sacrificed network integrity. XG, leveraging its rigid chain conformation, significantly increased the storage modulus by forming an interpenetrating network through chain entanglements. This comparison clearly reveals that the charge properties, chain rigidity, and gelation mechanism of polysaccharides collectively determine their role in composite networks. In a double-network construction, Luo et al. [63], inspired by biological fascial structures, fabricated a double-network hydrogel using soy protein amyloid fibrils and SA via Ca^2+^ crosslinking and compression molding, achieving a tensile strain of 91.2% under 2% SA conditions. Zhao et al. [64] constructed an interpenetrating network based on TGase-crosslinked SPI and cationically crosslinked sanxan gum. At 0.6% sanxan gum, the network was uniform and porous, with a freeze–thaw syneresis rate of only 16.5%. Khan et al. [65], addressing the issue of poor network integrity due to molecular binding competition in multi-component systems, proposed a “pre-complexation” strategy: first complexing microcrystalline cellulose with tannic acid before introducing it into the SPI matrix. The implementation of this strategy increased the gel’s water-holding capacity and strength to 57.8% and 19.3 g, respectively, with a bound water proportion of 97.9%, effectively avoiding binding competition between components.

**Figure 1:**
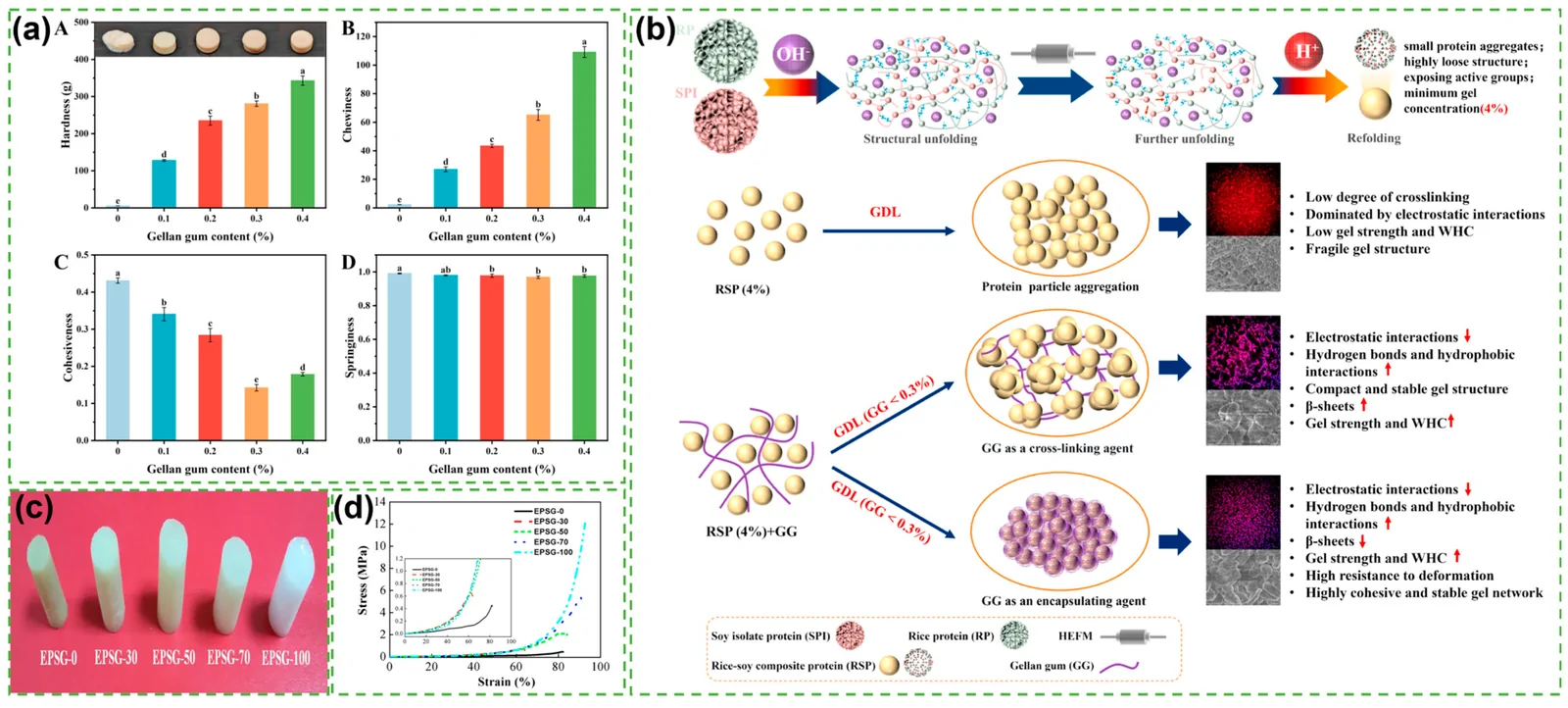
(**a**) The appearance and hardness (A), chewiness (B), cohesiveness (C), and springiness (D) of rice–soy composite protein gels with different GG concentrations. The different letters indicated significant differences (*p* < 0.05). (**b**) Potential mechanisms of GG in acid-induced gelation of rice–soy composite protein modified by HEFM-alkali-driven pretreatment. Reproduced with permission from reference [68]. Copyright 2026, Elsevier. (**c**) Photographs of the EPSG-n hydrogels. (**d**) Compressive stress–strain curves of the EPSG-n hydrogels. Reproduced with permission from reference [73]. Copyright 2022, Frontiers Media S.A.

The core concept of double-network design lies in the efficient dissipation of energy under stress through the interpenetration of a brittle first network and a flexible second network. Wang et al. [34] constructed a secondary composite network by combining a dialdehyde sodium alginate-crosslinked SPI network with a Ca^2+^ ion-crosslinked network. This enabled the gel to achieve a compressive strength of 28 kPa at 50% strain, 4.24 times that without Ca^2+^, perfectly illustrating the toughening mechanism of “sacrificial bonds dissipating energy.” Xu et al. [66] further combined Schiff base bonds with catechol-Fe^3+^ coordination bonds to construct a dual-dynamic crosslinked hydrogel, revealing that the dynamic bond content is the core lever for regulating gelation rate, mechanical performance, and self-healing ability. Introducing nanomaterials extends the scale of reinforcement from the molecular to the nanoscale. Chen et al. [67] introduced positively charged chitin nanofibers into SPI gels and found that they acted not only as inert fillers but also as efficient gelation promoters. The final compressive stress was approximately twice that of pure SPI gels, forming a composite structure akin to reinforced concrete. Dai et al. [68] discovered that only 0.4% guar gum could increase the hardness and chewiness of composite protein gels by approximately 61 and 42 times (Figure 1a), respectively. The remarkable enhancement was attributed to guar gum’s unique role-switching behavior: acting as a crosslinker at low concentrations and transitioning to a filler at high concentrations. The potential mechanism of guar gum in rice bean complex protein acid-induced gel is shown in Figure 1b. Fu et al. [69] revealed the profound impact of crosslinking sequence on gel structure: pre-heating SPI to unfold its structure exposed more reactive amino groups, significantly enhancing the degree of Schiff base crosslinking with dialdehyde carboxymethyl cellulose. The observed crosslinking behavior exemplifies what we define as a “conformational activation–chemical locking” strategy, where a physical stimulus (heat) is first used to activate the protein chains by exposing reactive sites, followed by a chemical crosslinking step to “lock” the activated conformation into a permanent and robust network. This two-step approach ensures that the maximum number of reactive groups are available for crosslinking, resulting in a denser and more stable network.

When application scenarios demand extreme multifunctionality, such as high strength–toughness, strong adhesion, antibacterial properties, and long-term stability, biomimetic strategies provide powerful design guidelines. Ma et al. [70] used dynamic coordination crosslinking between pyrogallol and borax to prepare PAM-SPI-P/B hydrogels, achieving 760% elongation, 1.25 MPa compressive strength, self-healing, and strong, repeatable adhesion. Zhang et al. [71], inspired by mussels and oysters, in situ deposited silver nanoparticles onto tannic acid-coated boron nitride nanosheets, yielding hybrid gels that integrate redox-initiated self-catalytic polymerization and multifunctionality. Cold-press shear strength increased by 818.2%, and the gels possessed long-term antibacterial and flame-retardant properties. Additionally, physical field-assisted processing can regulate the microstructure of composite networks without changing formulations. Sun et al. [72] combined ultrasonication with Fe^2+^ coordination crosslinking, achieving an Fe^2+^ encapsulation efficiency of 82.5%. Zhao et al. [73] introduced polyvinyl alcohol (PVA) into SPI *via* freeze–thaw cycles (Figure 1c), enabling the gel to achieve a compressive strength of 5.38 MPa at 89.5% water content (Figure 1d). Yan et al. [74] emphasized that when preparing corn fiber gum (CFG)–SPI double-network gels *via* laccase oxidation and heat treatment, synchronously regulating CFG, SPI, and pH was necessary to lock in the optimal performance window, underscoring the importance of systems engineering thinking in composite strategies.

A composite strategy that synergistically integrates multiple crosslinking mechanisms represents the current state of the art in soy protein-based hydrogel design, achieving properties that surpass those of any single approach. The bio-tissue-inspired dual-network concept is particularly effective at simultaneously enhancing strength and toughness through energy dissipation mechanisms. However, the complexity of multicomponent systems poses challenges for reproducibility and predictability, as interactions among components are often non-additive and highly sensitive to formulation and processing conditions. “Pre-composite” strategies and fine-tuned control of crosslinking sequences offer promising avenues for addressing these challenges. Future developments should focus on establishing quantitative structure–property relationships to enable predictive design rather than empirical optimization.

### 3.5. A Comparative Analysis of Cross-Linking Strategies

The four cross-linking strategies described above differ fundamentally in terms of their mechanisms, advantages, limitations, and suitability for various applications. Physical cross-linking is the mildest and most cost-effective method; it preserves protein bioactivity and avoids the use of toxic reagents, but the resulting hydrogels have relatively low mechanical strength and stability, making them suitable for food applications that require mild conditions. Chemical cross-linking provides the highest mechanical strength and stability through permanent covalent bonds but may compromise biocompatibility due to cytotoxic cross-linking agents or harsh reaction conditions; it is best suited for environmental and agricultural applications where mechanical robustness is critical. Enzymatic cross-linking offers excellent specificity and biocompatibility under physiological conditions, making it an ideal choice for biomedical and food applications, but it is limited by slow kinetics, high cost, and substrate dependence. Hybrid strategies achieve balanced performance through the synergistic combination of multiple mechanisms but face challenges in reproducibility, scalability, and predictive design due to system complexity.

From a translational perspective, physical and enzymatic cross-linking are typically preferred for food applications due to their “clean label” appeal and regulatory acceptability. Although chemical cross-linking offers superior mechanical properties, it faces regulatory barriers in food and biomedical applications due to concerns about residual cross-linking agents and modification byproducts. Composite strategies, despite their superior performance, pose the greatest challenges for process control and quality assurance, as interactions among multiple components must be carefully balanced to ensure consistent performance. Therefore, the choice of crosslinking strategy should be based on the specific requirements of the target application and involve a careful weighing of trade-offs between performance, safety, cost, and scalability.

To systematically integrate the key information from the four crosslinking strategies above, Table 3 summarizes and compares the crosslinking mechanisms, gel conditions, and key characteristics. On this basis, Table 4 further employs a semi-quantitative evaluation system to comprehensively compare strategies across dimensions such as mechanical strength, network stability, biocompatibility, scalability, cost, and process complexity, providing researchers with a reference for selecting suitable crosslinking pathways for specific application scenarios.

**Table 3:** Comparative Summary of Crosslinking Strategies for Soy Protein Hydrogels.

Crosslinking Strategy	Mechanism	Typical Conditions/Agents	Key Hydrogel Properties	Representative Applications	References
Physical	Non-covalent interactions (hydrophobic, H-bonding, electrostatic, ionic)	Heat, pH-shifting, ions (Ca^2+^), ultrasound, high pressure	Advantages: Mild, biocompatible, reversible; Limitations: Low mechanical strength, poor stability	Food, cell encapsulation	[33,35,37,38,39,40,41,42,43]
Chemical	Covalent bonds (Schiff base, Michael addition, etc.)	Glutaraldehyde, oxidized polysaccharides, genipin, EDC/NHS	Advantages: High strength, stability, tunable degradation; Limitations: Potential cytotoxicity, irreversible	Environmental remediation, drug delivery	[30,31,32,44,45,46,47,48,49,50,51,52,53]
Enzymatic	Isopeptide bonds, disulfide bonds	Transglutaminase (TGase), laccase, tyrosinase	Advantages: High specificity, biocompatible, mild conditions; Limitations: Slow kinetics, high cost, substrate-dependent	Biomedicine, food, smart materials	[36,54,55,56,57,58,59,60,61]
Hybrid	A combination of two or more of the above	Double network (DN), IPN, nanocomposite	Advantages: Synergistic properties (high strength, toughness, responsiveness); Limitations: Complex preparation, potential for non-additive effects	Wound healing, flexible sensors, high-performance materials	[34,62,63,64,65,66,67,68,69,70,71,72,73,74,75,76,77,78,79,80]

**Table 4:** Qualitative Comparison of Crosslinking Strategies for Soy Protein Hydrogels Based on Key Performance Criteria.

Criteria	Physical	Chemical	Enzymatic	Hybrid
Mechanical Strength	Low	High	Moderate	Very High
Biocompatibility	Excellent	Moderate	Excellent	Good
Scalability	High	High	Low	Low
Cost	Low	Moderate	High	High
Regulatory Acceptance	High	Low	High	Moderate
Stimuli-Responsiveness	High (pH, temp, ionic)	Low	Low	High

### 3.6. Structure–Property Relationships in Soy Protein Hydrogels

Understanding the relationships between molecular structure and macroscopic properties is essential for the rational design of SPHs with tailored performance. This section synthesizes current knowledge on key structure–property relationships.

1. Molecular Unfolding and Network Topology

The extent and manner of protein unfolding during gelation profoundly influence network topology. Partial unfolding exposes hydrophobic groups and reactive thiol groups while retaining some of the native structure, resulting in networks with a mixed α-helix/β-sheet content and moderate mechanical properties [81]. Extensive unfolding promotes widespread hydrophobic aggregation and disulfide cross-linking, producing denser, stiffer networks, but may come at the expense of water-holding capacity [82]. The 7S fraction has lower thermal stability and is more prone to unfolding, tending to form chain-like and branched aggregates, whereas the 11S fraction is more stable and forms granular aggregates, which contributes to network rigidity.

2. Secondary Structure and Mechanical Properties

The secondary structure composition of soy protein, particularly its β-sheet content, is directly related to the mechanical properties of hydrogels [83]. A higher β-sheet content is typically associated with increased gel stiffness and strength, as β-sheets form an extensive network of intermolecular hydrogen bonds that reinforce the gel matrix [84]. Conversely, a higher α-helix content is associated with greater flexibility and ductility. However, excessive β-sheet aggregation can lead to brittleness, underscoring the importance of a balanced secondary-structure composition.

3. Crosslinking Density and Swelling Behavior

Crosslinking density is the primary factor determining the swelling behavior of hydrogels. A higher crosslinking density restricts the mobility of polymer chains, reduces the effective free volume available for water absorption, and results in a lower equilibrium swelling ratio [85]. This relationship is described by the Flory–Rehner theory, which characterizes the balance between osmotic pressure (which drives swelling) and elastic recoil forces (which resist swelling). In soy protein-based hydrogels, crosslinking density can be controlled by adjusting the crosslinking agent concentration, reaction time, or enzyme dosage. However, this relationship is nonlinear: excessive crosslinking may mask functional groups and reduce effective pore size, thereby impairing swelling and the release of encapsulated active substances.

The relationship between crosslinking density and material performance involves a fundamental trade-off that is particularly evident in adsorption and controlled-release applications. On one hand, higher crosslinking density enhances mechanical strength, network stability, and resistance to deformation. On the other hand, excessive crosslinking reduces the effective pore size and porosity, shields functional groups (e.g., carboxyl, amino) that are essential for adsorption or binding of active molecules, and restricts chain mobility, thereby reducing adsorption capacity and swelling ratio. This trade-off, which is rooted in the Flory–Rehner theory describing the balance between osmotic pressure (driving swelling) and elastic recoil forces (resisting swelling), has been observed across multiple SPH systems. Similarly, in controlled-release fertilizer applications, excessive crosslinking can reduce the release rate of nutrients to suboptimal levels. This trade-off underscores the importance of precisely tuning crosslinking density to achieve the optimal balance between mechanical integrity and functional performance for each specific application.

4. Pore Structure and Mass Transport

The pore size and porosity of soy protein-based hydrogel networks directly influence the diffusion of nutrients, drugs, and metabolites. Gels with large pore sizes (>100 nm) allow macromolecules to diffuse freely and facilitate cell infiltration, making them suitable for tissue-engineering scaffolds. Gels with small pore sizes (<50 nm) restrict macromolecular diffusion, providing a sustained-release profile for embedded therapeutic agents [86]. The pore structure is determined by the aggregation state of the protein structural units: chain-like aggregates produce fibrous networks and large pores, while particle-like aggregates produce denser networks and small pores. Processing parameters such as heating rate, pH, and ionic strength can be used to control aggregate morphology, thereby regulating pore structure [87].

5. Rheological Behavior and Molecular Interactions

The rheological properties of soy protein-based hydrogels—including the storage modulus (G’), loss modulus (G″), and gelation kinetics—reflect the underlying landscape of molecular interactions. Hydrogels dominated by covalent cross-links exhibit frequency-independent behavior and high G’ values, indicating permanent network connections. Hydrogels dominated by noncovalent interactions exhibit frequency-dependent behavior and lower G’ values, reflecting the dynamic nature of these reversible bonds [88]. The gelation point marks the sol–gel transition and is sensitive to protein concentration, temperature, and crosslinking agent concentration [89]. Understanding these rheological characteristics enables the prediction of gel behavior under various processing and application conditions.

## 4. Major Application Fields of Soy Protein Hydrogels

Leveraging their unique biocompatibility, tunable physicochemical and mechanical properties, and inherently green and sustainable nature, soy protein hydrogels have rapidly transitioned from fundamental research to diverse practical application scenarios. Their value lies not only in substituting for traditional materials but also in creating products with novel functionalities. This chapter examines the progress of research on soy protein hydrogels in biomedicine, food and nutrition, environment/agriculture, and emerging fields.

### 4.1. Biomedicine

The application of soy protein hydrogels in the biomedical field has grown rapidly. The inherent biological advantages of soy protein drive this surge. These include abundant cell adhesion sites (e.g., RGD-like sequences), low immunogenicity, controllable enzymatic degradability, and metabolizable degradation products (amino acids). These traits endow SPHs with the potential to mimic the extracellular matrix and serve as carriers for bioactive molecules.

In tissue engineering, the core mission of soy protein hydrogels is to provide cells with a biomimetic three-dimensional microenvironment. For bone tissue, the mainstream strategy involves combining hydrogels with inorganic ceramics to mimic the organic–inorganic hybrid structure of natural bone. Alesaeidi et al. [90] introduced hydroxyapatite into a soy protein/alginate network, elevating the storage modulus from 10^3^ Pa to 10^4^ Pa, with no cytotoxicity toward osteoblasts. Zulkifli et al. [91] used oil palm empty fruit bunch waste as a raw material and combined glutamic acid crosslinking and freeze-drying to prepare SPI/CMC porous scaffolds. These scaffolds could slowly degrade within 35 days in simulated body fluid and induce calcium phosphate deposition, embodying the sustainable concept of “waste-derived raw material—green processing.” Cartilage repair faces a core contradiction: long-term mechanical stability requires slow degradation, whereas native soy protein degrades relatively quickly in vivo due to matrix metalloproteinases. Using crosslinkers such as genipin to delay degradation requires careful consideration of their impact on cell viability. Skin regeneration demands more comprehensive biological functions. Liu et al. [75] found that in 3D-printed scaffolds, soy protein imparted mechanical properties, while soy peptides significantly promoted angiogenesis. Li et al. [92] co-blended silk cellulose with SPI loaded with quercetin, significantly accelerating epidermal regeneration, collagen deposition, and angiogenesis in infected burn wound models.

In wound management and hemostasis, next-generation dressings are evolving from passive coverage to active promotion of healing. Varshney et al. [93] prepared SPI porous scaffolds *via* low-temperature gelation technology with approximately 90% porosity and a swelling ratio exceeding 1200%. Leveraging electrostatic capture of red blood cells by surface carboxyl and amino groups of the protein, they demonstrated superior hemostatic ability compared to traditional gauze. Zhao et al. [94] prepared composite gels by crosslinking hydroxypropyl chitosan with SPI using epichlorohydrin, achieving a hemostasis time of only 75 s in a New Zealand rabbit liver hemostasis model. To address chronic infected wounds, Huang et al. [76], inspired by mussels, constructed a TA-PAM-SPI double-network adhesive hydrogel with a tensile strain of 932%, integrating self-healing, antibacterial properties, and repeatable adhesion. Zhou et al. [95] directly addressed the challenge of radiation-induced skin injury by designing an SPI-SH-GA@PGO biological gel that reduces reactive oxygen species (ROS) and NLRP3 expression via the Wnt3a/β-catenin pathway, thereby elevating functionality from “passive filling” to active “immune-regeneration regulation.” For light-responsive platforms, He et al. [96] used black phosphorus grafted with clarithromycin to achieve synergistic photothermal/photodynamic antibacterial effects, achieving an inhibition rate exceeding 99% and shortening the healing time of infected wounds by 4 days. Liu et al. [97] developed a UV-polymerized self-adhesive hydrogel from thiol-grafted SPI, demonstrating significant wound-healing-promoting effects in diabetic chronic wound models.

**Figure 2:**
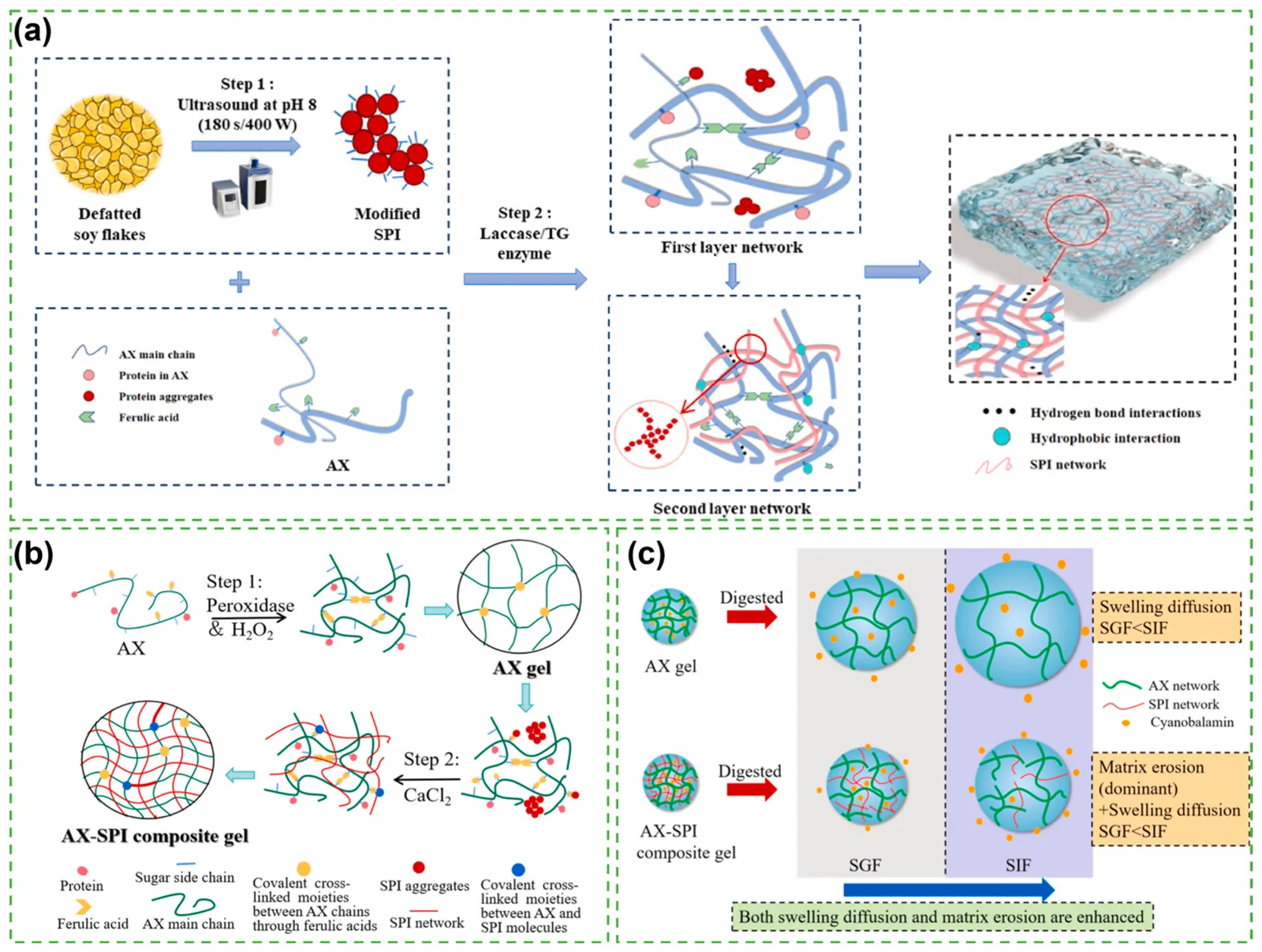
(**a**) Model diagram of the formation mechanism of the SPI-AX DN hydrogel. Reproduced with permission from reference [77]. Copyright 2025, Elsevier. (**b**) Schematic diagram of the formation process of the composite gel. (**c**) Release mechanism of cyanocobalamin from AX gel and composite gel under gastrointestinal conditions. Reproduced with permission from reference [98]. Copyright 2025, Elsevier.

In drug delivery, the multi-responsive nature and tunable degradation kinetics of soy protein hydrogels make them a natural platform for intelligent delivery. The ability of soy protein to contract at its isoelectric point in gastric acid and swell in the intestine enables targeted oral delivery. Tao et al. [77] constructed a pH-responsive double-network hydrogel from ultrasonicated SPI and arabinoxylan, achieving a riboflavin release efficiency of 98.04% with minimal gastric losses. The model diagram of the formation mechanism is shown in Figure 2a. Yan et al. [98] employed a dual-induction strategy of peroxidase crosslinking and Ca^2+^ crosslinking to prepare AX-SPI composite gels, achieving the dual goal of gastric protection and rapid intestinal release. The formation process of composite gel and the release mechanism of cobalamin from AX gel and composite gel under gastrointestinal conditions are shown in Figure 2b,c. To enhance the bioavailability of poorly soluble drugs, Cui et al. [90] prepared resveratrol-loaded SPI–dipotassium glycyrrhizinate nanocomplexes, thereby increasing the drug’s apparent solubility by a factor of 482.53. In animal experiments, the bioavailability was 5.17 times that of the free drug. In cancer therapy, Vialea et al. [99] combined SPI hydrogels with doxorubicin-loaded liposomes to develop implantable materials to combat glioblastoma recurrence post-surgery. Their targeting ability against GB cells was validated in 3D-bioprinted models.

For tissue adhesion, achieving firm adhesion at wet interfaces is one of the most challenging aspects of hydrogels. Argenziano et al. [100], inspired by mussel wet adhesion mechanisms, reacted soy protein with plant polyphenols, such as caffeic acid, chlorogenic acid, and gallic acid, under alkaline aerobic conditions, yielding fully natural tissue adhesives. Among them, the SPI/gallic acid system remained stable underwater for 15 days and exhibited biocompatibility, hemocompatibility, and wound-healing-promoting activity.

To provide a structured overview of the biomedical applications of SPHs, Table 5 summarizes the key performance indicators across different application scenarios and design strategies. It is evident that mechanical properties vary widely depending on crosslinking strategy and composite formulation: physically crosslinked hydrogels typically exhibit compressive strengths in the range of 5–50 kPa, whereas chemically or hybrid-crosslinked systems can reach 100–500 kPa or higher. Similarly, degradation times range from days (for physically crosslinked systems) to months (for genipin-crosslinked or nanocomposite systems), reflecting the fundamental trade-off between mechanical robustness and biodegradability. Notably, hydrogels designed for bone tissue engineering prioritize high stiffness and slow degradation, while wound dressings emphasize swelling capacity, antibacterial activity, and moderate degradation to match tissue regeneration rates. These comparisons underscore that there is no single ‘optimal’ SPH formulation; rather, the design must be tailored to the specific requirements of each application scenario, with careful consideration of the trade-offs between mechanical performance, degradation kinetics, and biological functionality.

**Table 5:** Biomedical Applications—Summary of Key Performance Indicators.

Application	Design Strategy	Compressive Strength (kPa)	Swelling Ratio (%)	Degradation Time	Key Functional Feature	Refs
Bone Tissue Engineering	SPI/alginate + HAp	~10^4^	~200	35 days	Osteoconductivity, cell proliferation	[90]
Cartilage Repair	Genipin-crosslinked SPI	50–200	150–300	4–8 weeks	High mechanical stability	[91]
Skin Regeneration	Silk fibroin/SPI + quercetin	30–80	800–1200	2–4 weeks	Angiogenesis, antibacterial	[75,92]
Wound Dressing	SPI porous scaffolds	5–20	>1200	1–3 weeks	Hemostasis, exudate absorption	[93,94]
Chronic Wound	TA-PAM-SPI DN hydrogel	100–300	500–1000	2–6 weeks	Self-healing, antibacterial, adhesive	[76,95]
Drug Delivery (Oral)	Ultrasonicated SPI-arabinoxylan	50–150	200–500	pH-responsive	Gastric protection, intestinal release	[77,98]
Cancer Therapy	SPI + DOX liposomes	20–50	300–600	4–8 weeks	Targeted release in tumor microenvironment	[99]
Tissue Adhesive	SPI + gallic acid (oxidative)	10–30	N/A	15 days (underwater)	Wet adhesion, biocompatibility	[100]

However, for clinical applications—particularly implantable and parenteral applications—the immunogenicity of soy protein is a critical issue that must be carefully evaluated. Certain antigenic epitopes present in soy protein may trigger allergic reactions or immune responses in some individuals [101]. Several processing strategies have been shown to effectively reduce the immunoreactivity of soy protein. A combination of enzymatic hydrolysis and Lactobacillus fermentation can produce hypoallergenic soy protein microgels by destroying allergenic epitopes. Fermentation-induced gel matrix formation can also enhance intestinal barrier integrity and promote the degradation of specific allergens [102]. Furthermore, heat treatment, high-pressure treatment, covalent conjugation with polyphenols, the Maillard reaction, and microbial fermentation have all been reported to reduce the antigenicity of soy protein by destroying antigenic epitopes or promoting protein degradation [103]. During hydrogel formation, the cross-linked three-dimensional network structure may further modulate immunoreactivity by altering the degradation kinetics and epitope exposure of proteins during physiological digestion. However, research on the immunogenicity of soy protein-based hydrogels intended for long-term implantation or repeated parenteral administration remains insufficient. This is because immunogenicity may change after proteins undergo chemical cross-linking or severe physical denaturation—sometimes being masked, and other times potentially enhanced due to the exposure of new epitopes. Therefore, before use in implantable materials or injectable formulations, specific formulations must undergo rigorous in vitro testing of cellular immune responses and in vivo animal studies. Currently, effective strategies for reducing the immunogenicity of SPHs include the enzymatic removal of immunodominant fragments, grafting with “masking” polymers such as polyethylene glycol (PEG), or constructing dense composite networks to physically shield antigenic sites [104]. Future research should prioritize systematic in vivo immunogenicity studies to establish a safety profile for the clinical translation of soy protein-based hydrogels.

SPHs hold tremendous potential in the biomedical field, but their clinical translation still faces significant challenges. In addition to the immunogenicity issues mentioned earlier, there remains a lack of data on the compatibility between long-term in vivo degradation behavior and mechanical properties, the impact of sterilization methods on gel structure and biological activity, and systematic biocompatibility and toxicology evaluation data that comply with medical device regulations. Future research should shift from a laboratory-performance-oriented approach to one centered on meeting the standardization requirements of specific clinical application scenarios, while strengthening collaboration with regulatory science.

### 4.2. Food and Nutrition

The application of soy protein hydrogels in the food field has a long history, from traditional tofu to modern plant-based meat products. Its core has always revolved around constructing three-dimensional networks to immobilize water, simulate fat mouthfeel, and serve as a texture carrier. With the rise of the plant-based food wave and the demand for personalized nutrition, this field has transitioned from a coarse “capable of gelation” stage to an era of fine control characterized by “precise structural design—intelligent manufacturing—targeted delivery.” The role of soy protein hydrogels has correspondingly shifted from passive texture filler to an active functional delivery platform and structural manufacturing ink.

**Figure 3:**
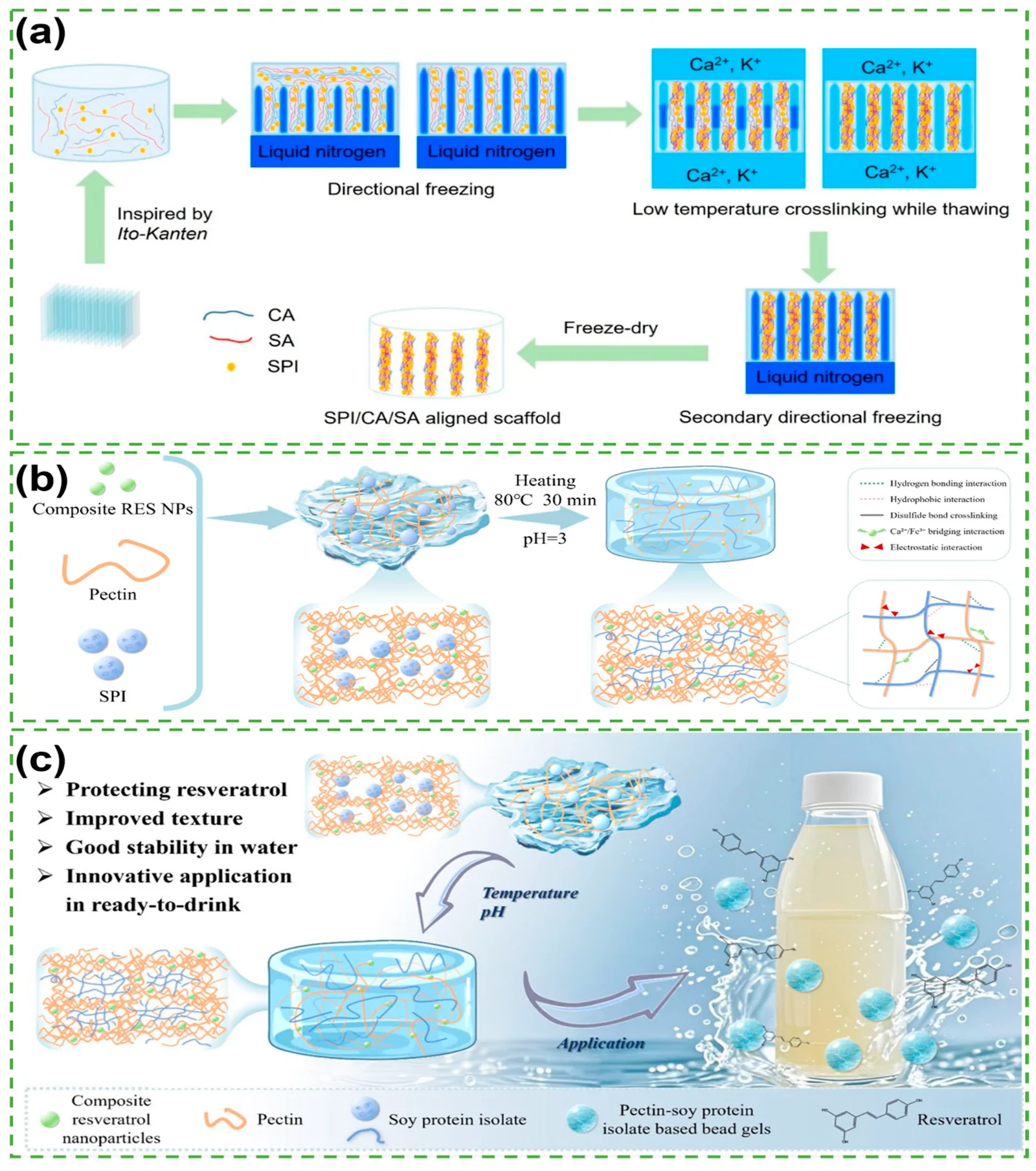
(**a**) Schematic illustration of the fabrication of the aligned scaffold. Reproduced with permission from reference [78]. Copyright 2025, MDPI. (**b**) Schematic of the double-network gel formation mechanism. (**c**) Schematic of double-network gel preparation and application. Reproduced with permission from reference [117]. Copyright 2026, Elsevier.

The core competitiveness of plant-based meat products lies in their ability to replicate the fibrous structure and chewiness of animal-derived foods, whereas traditional heat-induced soy protein gels are often isotropic and disordered. The combined use of directional freezing and salting-out techniques provides a novel solution to break through this limitation. Yang et al. [105] formed oriented fibers from soy protein and konjac glucomannan *via* directional freezing, subsequently locking the network by salting out. The resulting anisotropic hydrogel achieved a maximum stress of 1.29 MPa, with a friction coefficient and water-holding capacity comparable to those of real meat. From the perspective of cultured meat, Xia et al. [78] used liquid-nitrogen directional freezing to prepare SPI–carrageenan–alginate anisotropic scaffolds (Figure 3a). After seeding with C2C12 cells, the cells differentiated along the scaffold orientation, thereby extending the application to the cultured meat frontier. In simulating complex multi-component structures, Liu et al. [106] collaboratively constructed connective tissue-mimicking hydrogels using SPI, corn starch, KGM, and seaweed powder. When polysaccharide concentration increased from 1% to 3%, hardness jumped from 316 g to 1827 g, and sensory scores were most similar to real connective tissue. Nie et al. [107] constructed high-internal-phase emulsion-based fat analogs using SPI and hydroxypropyl sulfated guar gum, achieving droplet sizes as small as 2.32 μm and demonstrating both flavor encapsulation and controlled release. Kim et al. [108] composited TGase-crosslinked fish gelatin with SPI and κ-carrageenan, producing boiled fish analogs with apparent surface fibers, whose texture could be flexibly adjusted with enzyme concentration. Notably, Zhang et al. [109] used SPI-KGM hydrogels as “saltiness carriers” for low-sodium surimi, promoting targeted sodium release during simulated chewing to enhance saltiness perception. The study demonstrates a novel hydrogel delivery strategy for solving the industry challenge of “salt reduction without compromising taste.

In the field of 3D food printing, soy protein hydrogels are considered ideal inks due to their shear-thinning behavior and rapid modulus recovery. Pan et al. [110] introduced golden needle mushroom polysaccharide into SPI hydrogels, achieving printing accuracy exceeding 99.7%. The polysaccharide promoted the α-helix-to-β-sheet transition *via* hydrogen bonding, thereby simultaneously reducing viscosity and enhancing network strength. Liu et al. [111] systematically investigated the influence of xanthan gum side chains on the printing performance of SPI composite gels. All formulations met the International Dysphagia Diet Standardization Initiative (IDDSI) framework, providing quantitative guidance for designing personalized therapeutic diets. Patil et al. [112] used SPI, barnyard millet flour, and guar gum as raw materials, employing citric acid crosslinking to prepare edible straws with a compressive strength of 9.1 MPa and complete biodegradation within 10 days, offering a plant-based alternative for single-use plastics.

In encapsulating and delivering functional factors, protecting sensitive substances like probiotics, vitamins, and polyphenols, and achieving controlled release are core objectives. For probiotic protection, Praepanitchai et al. [113] encapsulated *Lactobacillus plantarum* in sodium alginate–SPI mixed gel beads. At an SA: SPI ratio of 1:8, encapsulated cells survived a heat treatment at 72 °C for 90 s. Vrik et al. [114] achieved a high encapsulation efficiency of 95.95% using SPI composites prepared via multi-frequency ultrasound assistance, thereby protecting probiotics for at least 8 weeks. Liu et al. [115] constructed a hydrogel–oleogel bigel system that encapsulated probiotics in the oil phase with 90.3% efficiency. After 4 weeks of storage, viable cell count remained as high as 5.47 × 10^9^ CFU/g, with gastric survival rate exceeding 87%. For oral nutrient delivery, Yan et al. [116] constructed a pH-responsive double-network hydrogel from corn fiber gum–SPI. It exhibited low swelling in simulated gastric fluid, protecting riboflavin, and substantial release in simulated intestinal fluid, following non-Fickian diffusion. Zhou et al. [117] constructed a pectin–SPI double-network gel encapsulating resveratrol (Figure 3b,c), with retention exceeding 92% after 28 days of storage at room temperature. When made into bead gels and added to beverages, the structure remained intact for over 10 h, providing a new paradigm for hydrophobic nutrient fortification in transparent beverages.

For food applications, the performance metrics of SPHs are evaluated against different benchmarks depending on the intended use. Table 6 summarizes the key parameters for major food applications. Plant-based meat analogs require anisotropic structures with tensile strengths approaching 1–2 MPa to mimic the fibrous texture of animal muscle, which has been achieved through directional freezing and salting-out strategies. Encapsulation systems for probiotics demand high encapsulation efficiency (>85%) and gastric survival rates (>80%), while 3D food printing requires shear-thinning behavior with rapid modulus recovery and printing accuracy exceeding 95%. Notably, the ‘beany flavor’ issue remains a significant sensory barrier across all food applications, although the crosslinked hydrogel network may partially mitigate volatile release through physical encapsulation. The diversity of these performance requirements highlights the versatility of SPHs as a food material platform, while also emphasizing that formulation and processing must be carefully optimized for each specific food application.

**Table 6:** Food and Nutrition Applications—Summary of Key Performance Indicators.

Application	Design Strategy	Tensile/Compressive Strength (MPa)	Water Holding Capacity (%)	Encapsulation Efficiency (%)	Key Functional Feature	Refs
Plant-Based Meat Analog	Directional freezing + salting-out	~1.29 (tensile)	80–95	N/A	Anisotropic fibrous structure	[105]
Cultured Meat Scaffold	Liquid-N2 directional freezing	0.5–1.0	85–92	N/A	Cell alignment along scaffold	[78]
Fat Analog	HIPE emulsion gel	0.01–0.05	70–85	N/A	Flavor encapsulation, mouthfeel	[107]
3D Food Printing	SPI + polysaccharide (e.g., FVP)	0.02–0.10	75–90	N/A	Shear-thinning, accuracy >99%	[110,111]
Edible Straws	Citric acid-crosslinked SPI/guar gum	~9.1 (compressive)	N/A	N/A	Biodegradable (<10 days), plastic alternative	[112]
Probiotic Encapsulation	Alginate–SPI beads	N/A	N/A	85–96%	Gastric survival >80%, 8-week storage stability	[113,114,115]
Nutrient Delivery (Riboflavin)	CFG-SPI DN hydrogel	0.1–0.5	60–80	70–90%	pH-responsive, gastric protection, intestinal release	[116]
Hydrophobic Nutrient (Resveratrol)	Pectin–SPI DN hydrogel	0.05–0.20	70–85	>92%	Sustained release in beverages, room-temperature stability	[117]

A significant sensory issue in the application of soy protein-based foods is the characteristic “beany odor,” which may limit consumer acceptance. This off-flavor primarily stems from volatile carbonyl compounds—such as hexanal and hexanol—generated by the oxidation of polyunsaturated fatty acids (particularly linoleic acid and linolenic acid) catalyzed by lipoxygenases; these compounds are described as having grassy, raw, and oily flavors. The levels of total volatiles and bean-like odor compounds have been shown to correlate positively with lipoxygenase activity and unsaturated fatty acid levels [118,119]. Various strategies—including heat, alcohol, and acid treatments—have been developed to rapidly inactivate lipoxygenase, thereby reducing the bean-like odor in soy protein products. Studies indicate that heat treatment at temperatures above 65 °C significantly affects the release of off-flavors by altering protein structure, with higher temperatures generally reducing the release of volatiles [120]. The use of lipoxygenase-deficient soybean varieties has also been explored as a genetic approach to producing low-off-flavor soy products [121]. In the context of hydrogel formation, the cross-linked three-dimensional network may reduce the release of volatile compounds during consumption by physically encapsulating them, although further research is needed in this area. Encapsulating flavor compounds within a hydrogel matrix or incorporating masking agents is an additional strategy to improve the sensory acceptability of soy protein-based foods.

SPHs hold great promise for application in the food industry, but their commercialization faces multiple challenges related to sensory acceptance, regulatory compliance, and cost-effectiveness. Issues such as bean-like odor must be addressed at the source—through raw materials and processing methods. As food additives or contact materials, their safety assessments (including potential migration of cross-linking agents or nano-components) must comply with food safety regulations in various countries. Furthermore, compared with existing food colloids (such as gelatin and carrageenan), SPHs must offer clear advantages in cost, performance, or sustainability to gain widespread adoption in industry.

### 4.3. Environment and Agriculture

The molecular chains of soy protein are naturally rich in functional groups such as carboxyl, amino, and hydroxyl groups, making them ideal green building blocks for constructing environmentally functional materials. These groups can efficiently capture heavy metals and organic pollutants from water bodies through mechanisms such as electrostatic adsorption, chelation, and ion exchange. Meanwhile, the three-dimensional network of hydrogels endows them with excellent water absorption and retention capacity, demonstrating unique value for agricultural soil amendment and fertilizer-controlled release [122]. More importantly, soy protein hydrogels can be gradually degraded by environmental microorganisms into amino acids after use, thereby avoiding secondary solid waste problems associated with traditional synthetic resins or activated carbon and aligning with the core demands of a circular economy [123,124]. However, translating this potential into effective environmental remediation still requires systematically overcoming key bottlenecks, such as synergistic regulation of adsorption capacity, structural stability, and degradation rate.

The removal of heavy metals and organic dyes from aquatic environments is the primary application scenario for soy protein hydrogels in environmental applications. Their adsorption efficacy results primarily from the synergistic effects of multiple forces, such as electrostatic attraction and chelation. Regarding selective adsorption, Liu et al. [125] prepared composite hydrogels from SPI and polyvinylamine. In a coexisting system of Zn(II), Cd(II), and Pb(II), the selectivity coefficient for Cu(II) was as high as approximately 250. Moreover, the adsorbed Cu(II) could be reduced in situ to copper nanoparticles, regenerating the waste adsorbent into an efficient catalyst and realizing an “adsorption–in situ transformation” life-cycle design. In radioactive nuclide separation, Cao et al. [126] pioneered the use of SPI hydrogels for extracting uranium from seawater and simulated nuclear wastewater. They found that amino and carboxyl groups could form equatorial coordination structures with uranyl ions. The adsorption capacity reached 5.29 mg/g over 21 days in natural seawater, outperforming known unmodified natural biomolecular adsorbents. For organic dye removal, Cao et al. [127] constructed composite gels from waste fish-scale biochar, sodium alginate, chitosan, and SPI, which possess dual functionality: dye adsorption and a 100% antibacterial rate within 1.5 h. However, there exists an inherent contradiction between adsorption performance and mechanical stability. As elaborated in Section 3.6, the crosslinking density-modulated balance between network rigidity and pore accessibility creates a fundamental trade-off that must be carefully optimized for each application. Liu et al. [128] found that as the crosslinker dosage increased, the mechanical strength of SPI/alginate double-network hydrogels improved significantly, whereas the adsorption capacity decreased from 190 mg/g to 120 mg/g. This reveals a trade-off relationship in which excessive crosslinking sacrifices adsorption capacity by reducing the effective pore size and shielding functional groups.

**Figure 4:**
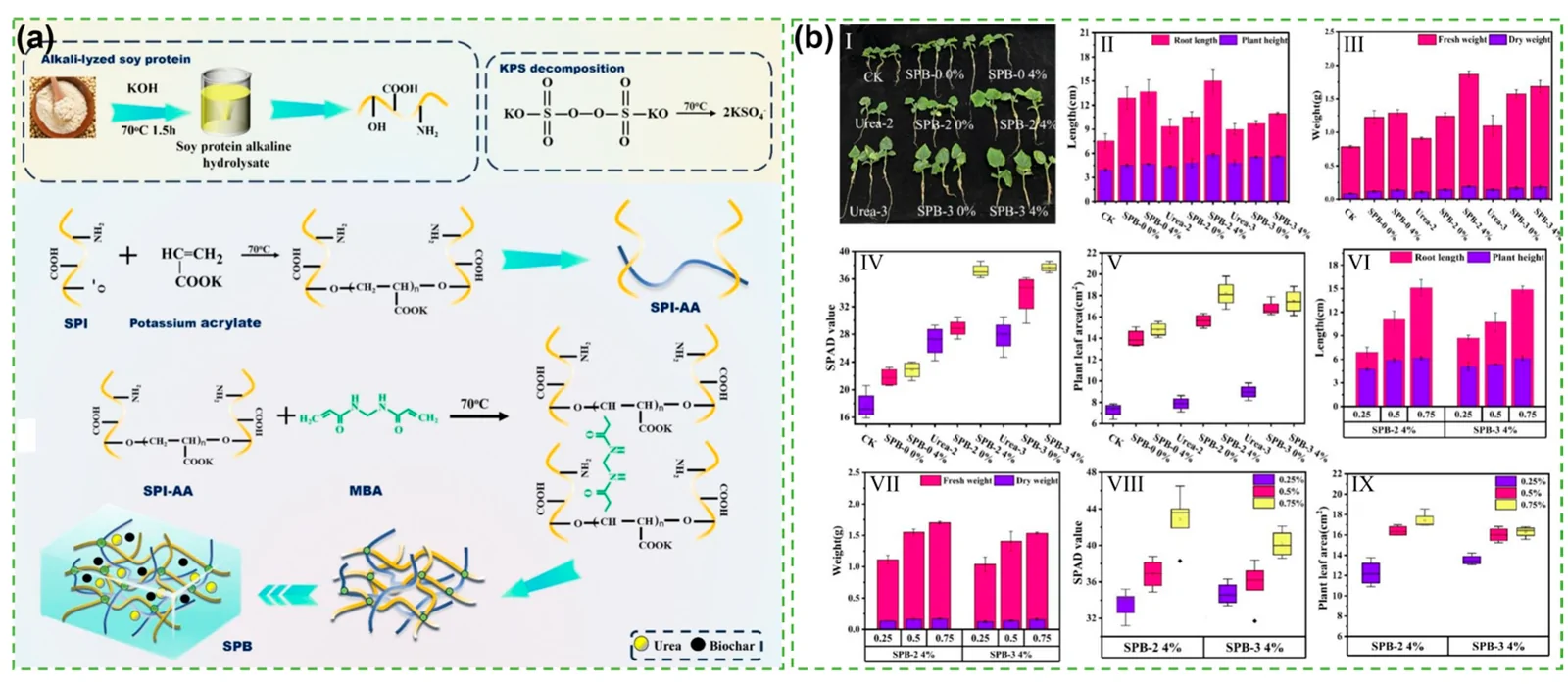
(**a**) Schematic diagram of SPB hydrogel preparation and internal cross-linking network. (**b**) Growth of cucumber in soil (5% addition) spiked with SPB-0/2/3–0%, SPB-0/2/3–4%, Urea-2/3 (consistent with SPB-2/3 urea content), and untreated soil (ck). After treatments, root length and plant height (II), dry and fresh weights (III), relative chlorophyll content (IV), leaf area (V); with different additions of SPB-2/3–4%, the cucumbers’ root length and plant height (VI), dry and fresh weights (VII), relative chlorophyll content (VIII), leaf area (IX) Reproduced with permission from reference [79]. Copyright 2024, Springer Nature.

In agriculture, the main value of soy protein hydrogels lies in soil water retention and fertilizer-controlled release. K.P. et al. [129] prepared controlled-release fertilizer hydrogels using PVA and SPI as base materials and citric acid as a green crosslinker. They released 74.1% urea over 28 days, retained water for up to 30 days, and achieved a soil degradation rate of 65% over 100 days, significantly improving crop growth performance. Liang et al. [79] introduced bamboo biochar to prepare SPI composite hydrogels with a swelling ratio of 222.3 g/g or higher. The schematic diagram of SPB hydrogel preparation and internal cross-linking network is shown in Figure 4a. Soil water retention capacity increased by 1.93 times, resulting in fresh weight, root length, and leaf area of cucumber seedlings increasing by 139.3%, 99.2%, and 149.5% (Figure 4b), respectively, while optimizing soil microbial community structure. Regarding micronutrient delivery, the Jiménez-Rosado team systematically studied the effects of different zinc salts on the superabsorbent capacity of soy protein matrices. They found that ionic strength significantly affected mechanical properties and water absorbency. ZnCO_3_ increased the water absorption capacity and formed more open-pore structures, whereas ZnSO4, when mixed with Zn-EDTA, showed no synergistic effect [130,131]. The team also developed low-cost controlled-release matrices (€0.50–2.00/kg) prepared *via* thermomechanical processing, increasing plant zinc assimilation by 60% while reducing irrigation water use by 33% [132]. For agricultural mulching films, Wang et al. [133] used oxidized sucrose as a green crosslinker and lignin as a nanofiller to prepare fully biodegradable SPI mulching films. Tensile strength increased to 8.45 MPa, and the films exhibited UV protection, heat and moisture retention, and slow urea release. Seed germination rate was higher than that with traditional polyethylene film.

In scalable preparation, Zhang et al. [134] developed a reactive spray-drying technique to prepare composite hydrogel microparticles from hydroxyethyl starch, SPI, ZIF-8, and epichlorohydrin, without the need for emulsifiers or additional drying steps. The maximum adsorption capacity for ciprofloxacin was 530 mg/g, driven by multiple mechanisms: electrostatic interactions, hydrogen bonding, π-π stacking, and pore adsorption. The demonstrated adsorption performance offers essential guidance for scaling soy protein hydrogel adsorbents from laboratory experiments to industrial production. Additionally, Cuadri et al. [135] prepared natural superabsorbent polymers by acylating soy protein with succinic anhydride. The water absorption rate reached 3398 wt%, and the material could be reused without loss of absorption capacity. Ma et al. [136] incorporated SPI into a sulfobetaine hydrogel network, thereby obtaining rechargeable, chlorine-based, bactericidal antifouling gels. Active chlorine content exceeded 400 ppm, capable of killing 6-log CFU of bacteria and viruses within 5 min.

The application of SPHs in environmental and agricultural fields highlights their ecological value; however, their practical deployment requires consideration of environmental durability, the risk of secondary pollution, and long-term ecological impacts. For example, when used as adsorbents, their selectivity, adsorption capacity, and regeneration performance in complex real-world water bodies require further validation; when used as agricultural carriers, the impact of their degradation products on soil microbial communities remains unclear. Future research should conduct more pilot-scale trials in real-world environments and establish a life cycle assessment (LCA) model to comprehensively evaluate their environmental benefits.

### 4.4. Smart Materials

If the food, biomedical, and environmental fields leverage the “natural attributes” of soy protein hydrogels, then the smart materials field embodies the ultimate exploitation of their “designability.” By combining soy protein with conductive materials, responsive molecules, or nano-functional fillers, researchers are transforming them from passively responsive soft matter into active platforms capable of sensing, actuation, and even energy storage, marking a transition in applications from “substitution-oriented” to “empowerment-oriented.”

**Figure 5:**
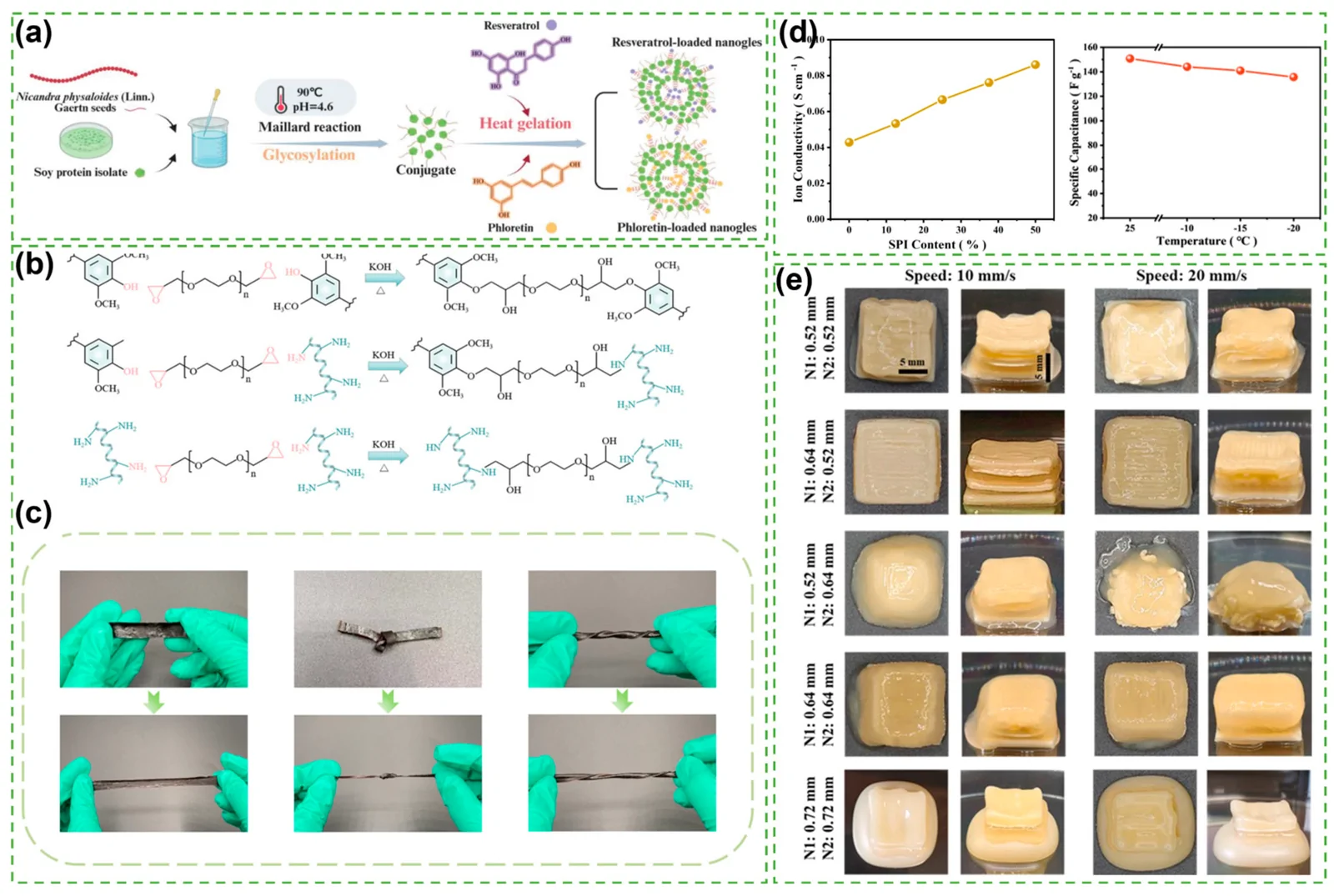
(**a**) The preparation process of NGs, Pht NGs, and Res NGs. Reproduced with permission from reference [141]. Copyright 2026, Elsevier. (**b**) Schematic diagram of Lig/SPI hydrogel reaction. (**c**) Excellent flexibility of Lig/SPI hydrogel. (**d**) Ionic conductivity of Lig/SPI-X and capacitance retention at different temperatures. Reproduced with permission from reference [152]. Copyright 2023, Springer Nature. (**e**) 3D printed sorghum protein (25%, *w*/*w*.) and soy protein (11%, *w*/*w*.) colloids with printing speeds of 10 mm and 20 mm per second, and nozzle sizes of 0.52, 0.64, and 0.72 mm. N1 and N2 refer to the nozzle sizes of extruder 1 and extruder 2, respectively. Reproduced with permission from reference [157]. Copyright 2025, Elsevier.

Active food packaging demands materials that not only provide physical barriers but also actively inhibit microbial spoilage and delay oxidative deterioration. Functional fillers represented by polyphenol–metal coordination networks endow films with unprecedented comprehensive performance. Wu et al. [137] blended konjac glucomannan and xanthan gum with an SPI–tannic acid–iron complex to produce active packaging films. They simultaneously achieved all-around performance: tensile strength of 20.30 MPa, UV shielding > 90%, soil degradation within 5 days, antioxidant activity > 80%, and antibacterial rate > 99%. The shelf life of blueberries was extended to 15 days. Cheng et al. [138] introduced tannic acid–iron metal–phenolic networks into corn starch/SPI films, increasing tensile strength by 96% and reducing water vapor permeability by 28%. In biomimetic superhydrophobic design, Sun et al. [139], inspired by the goose feather structure, incorporated L-cysteine–copper nanofibers and carnauba wax into an SPI matrix, thereby producing a superhydrophobic, antibacterial film with a contact angle of up to 152°. Tensile strength increased from 3.89 MPa to 11.65 MPa. Applied to salmon preservation, this extended the shelf life to 10 days. Regarding preservation carrier-form innovation, Shi et al. [140] incorporated a litsea cubeba essential oil nanoemulsion into SPI/alginate porous hydrogels for blueberry preservation, significantly reducing weight loss and decay over 9 days. Meng et al. [141] prepared SPI-pectin nanogels *via* a two-step Maillard dry-heating and self-assembly method (Figure 5a), simultaneously loading resveratrol and phloretin, and demonstrated both antioxidant and antibacterial activities. Particularly noteworthy, Wang et al. [142] developed biodegradable porous hydrogels with both cushioning protection and preservation functions. Initial loading could dissipate 70–78% of the deformation energy, with a dissipation rate of 51.52% after 200 cycles. The strawberry damage index inhibition rate was below 14%, indicating that functionality extended beyond chemical preservation to physical protection. For smart indication, Li et al. [143] encapsulated curcumin in SPI *via* a pH-driven method to produce 3D-printing inks, resulting in smart freshness labels that change from yellow to red as minced meat spoils.

Applying soy protein hydrogels to flexible electronics critically requires simultaneously achieving high ionic conductivity, excellent mechanical compliance, and environmental stability. Lv et al. [144] prepared conductive hydrogels from maleic anhydride-modified SPI crosslinked with acrylamide, achieving a conductivity of 2.8 S/m, capable of stably monitoring joint movements. To overcome the challenge of balancing mechanical and conductive properties, Deng et al. [80] leveraged synergistic effects of Ca^2+^/CO_3_^2−^ biomineralization and NaCl/Na_3_Cit salting-out, achieving a hydrogel with tensile strength of 1.40 MPa, toughness of 6.04 MJ/m^3^, sensor sensitivity (GF) of 3.18, and response time of only 0.33 s. Wang et al. [145] fused the Hofmeister effect with dynamic Schiff base crosslinking. H_2_PO_4_^−^ treatment increased gel hardness by 10.6 times and compressive strength by 13.0 times, while achieving a high conductivity of 6.49 S/m. Regarding extreme environmental tolerance, Ma et al. [146] prepared organohydrogels that remained flexible and conductive at −70 °C, with a conductivity of 0.63 S/m at −40 °C. Hu et al. [147] overcame salting-out embrittlement by co-introducing MgCl_2_ and Na_3_Cit, lowering the freezing point to −19.4 °C. In multifunctional integration, Li et al. [148] linked SPI, MXene, and a polymer network *via* hydrogen bonds, yielding a hydrogel with stretchability exceeding 800%, self-adhesiveness, injectability, and wide-temperature-range sensing capability. Wang et al. [149] used Zn^2+^ to form multiple crosslinking networks *via* coordination, hydrogen, and Schiff base bonds, endowing the hydrogel with both conductivity and antibacterial activity.

Soy protein is rich in heteroatoms, such as nitrogen and oxygen, making it an ideal biomass precursor for constructing supercapacitor electrodes and gel electrolytes. Li et al. [150] used SPI hydrogels as precursors for KOH-activated pyrolysis, thereby synthesizing N/O/S self-doped hierarchical porous carbons with a specific surface area of up to 1914.08 m^2^/g. The specific capacitance reached 320 F/g in a three-electrode system. For gel electrolytes, Wang et al. [151] increased ionic conductivity to 5.10 mS/cm by grafting acrylamide onto modified SPI. Assembled supercapacitors maintained 95% of their capacitance after 8000 cycles. Duan et al. [152] prepared electrolytes *via* covalent crosslinking of lignin and SPI (Figure 5b,c), achieving an ionic conductivity of 0.086 S/cm, retaining 90% of room temperature specific capacitance at −20 °C (Figure 5d).

The deep integration of advanced manufacturing technologies with soy protein hydrogels has enabled the development of customizable smart materials. Dorishetty et al. [153] prepared 3D-printable hybrid hydrogels *via* photochemical co-crosslinking of spherical soy protein and silk fibroin. After printing, pore size was 7.6 times higher than that of cast samples, supporting fibroblast proliferation. Ma et al. [154] prepared fully bio-based UV/thermal dual-curable 3D printing inks that combine high precision and excellent water resistance. In food printing, Arain et al. [155] found that SPI emulsions stabilized by low-methoxy pectin exhibited rapid thixotropic recovery and 99% water retention after Ca^2+^ crosslinking. At 30% oil fraction, high-resolution printing could be achieved. Xu et al. [156] proved that acacia polysaccharide enhanced the printing dimensional accuracy of SPI gels in a dose-dependent manner, involving multiple non-covalent synergies dominated by hydrophobic interactions. Barekat et al. [157] co-printed sorghum and soy protein gels using a dual-extruder (Figure 5e), thereby balancing the essential amino acid profile. Wang et al. [158] prepared biodegradable fire-extinguishing gels using SPI and gelatin as skeletons, achieving 81.2% soil degradation over 25 days. The extinguishing time was shortened by 29.79% compared with that of commercial sodium polyacrylate hydrogel.

SPHs have demonstrated unique advantages in the field of smart materials, such as flexible electronics and actuators; however, their performance (e.g., conductivity, response speed, and cycling stability) remains generally inferior to that of synthetic-polymer-based hydrogels. Long-term stability (e.g., dehydration and fatigue) and integration processes with rigid devices pose challenges for industrialization. Future breakthroughs will lie in the development of SPH-based smart systems with biomimetic hierarchical structures and stable performance, enabled by precise molecular design and advanced manufacturing technologies (such as 3D printing).

To help readers quickly grasp the diverse application prospects of soy protein hydrogels, Table 7 summarizes the functional requirements of the four major application fields, corresponding SPH design strategies, and benchmark performance indicators, and clearly outlines the current state of the art and the persistent gap.

## 5. Critical Challenges and Translation Barriers

Despite significant progress in SPH research, translating these materials from the laboratory to large-scale applications remains a formidable task. This is due to a confluence of challenges arising from the inherent complexity of the raw material, the sensitivity of the hydrogel network to external factors, and the stringent requirements of diverse application scenarios.

1. Raw Material Variability and Reproducibility

Soy protein isolate is a complex mixture whose composition varies depending on soybean variety, growing conditions, extraction methods, and processing parameters. The 7S/11S ratio, degree of denaturation, and trace-component content may vary significantly between batches, thereby affecting the reproducibility of the gelation process and the consistency of the final product’s performance. This batch-to-batch variability poses a major challenge for quality control and regulatory approval, particularly in biomedical applications that require consistent performance.

2. Oxidation and Long-Term Stability

Soy protein is susceptible to oxidation during processing and storage, particularly through lipid oxidation catalyzed by residual lipoxygenases or radical-mediated protein oxidation. Oxidation can lead to protein aggregation, cross-linking, and loss of functional groups, impairing gel formation and hydrogel performance [159,160]. Additionally, SPHs may undergo shrinkage due to dehydration, microbial degradation, or network restructuring during storage, affecting their functional lifespan. In the biomedical field, research on the association between long-term in vivo degradation behavior and immune responses remains insufficient.

3. Sensitization and Immunogenicity

As described in Section 4.1, soy protein contains multiple allergenic epitopes that may pose a risk to susceptible individuals. Although processing methods such as enzymatic hydrolysis, fermentation, and heat treatment can reduce sensitization, eliminating immunoreactivity remains challenging. For biomedical applications, particularly implantable devices, the potential for sensitization or adverse immune reactions requires thorough preclinical evaluation.

4. Sterilization

Sterilization is a key requirement for biomedical and many food applications, but most SPHs are sensitive to conventional sterilization methods. Autoclaving leads to protein denaturation and network collapse; ethylene oxide treatment may leave toxic residues; and γ-irradiation can induce cross-linking or degradation [161]. The development of soy protein-based hydrogel formulations compatible with sterilization or alternative sterilization methods is an important area of research.

5. Regulatory and Commercialization Barriers

For food applications, chemically modified or composite soy protein hydrogels require evaluation of cross-linking agent residues and nanoparticle migration in nanocomposites and may require new food approvals. For biomedical applications, soy protein-based hydrogels are classified as medical devices or combination products and require rigorous preclinical and clinical testing to demonstrate safety, efficacy, and manufacturing consistency. Furthermore, many composite or modification strategies that perform well in the laboratory may prove unfeasible during industrial scale-up due to high costs, thereby limiting commercial competitiveness. Life cycle assessment studies are needed to quantify the environmental benefits of soy protein-based hydrogels relative to petroleum-based alternatives and to identify opportunities for cost reduction. Simultaneously, the development of efficient, continuous SPHs forming processes is key to reducing costs and achieving industrialization.

## 6. Conclusions and Future Perspectives

### 6.1. Conclusions

This review systematically consolidates the complete chain of advancements in the field of soy protein hydrogels (SPHs), spanning from molecular design to functional applications. As an outstanding representative of sustainable bio-based materials, the successful construction of SPHs is rooted in the unique molecular endowments of soy protein, particularly the complementary characteristics of 7S and 11S globulins, its wealth of active functional groups, and inherent bioactive sequences. Through the synergy and innovation of diverse strategies—including physical, chemical, and enzymatic crosslinking, as well as composite/hybrid approaches—researchers can now precisely regulate the network structure of SPHs. Such regulation enables tunable mechanical properties, intelligent responsiveness, and additional functionalities such as antibacterial or conductive properties. These developments have significantly expanded the application boundaries of SPHs, successfully penetrating fields such as biomedicine, food and nutrition, environmental remediation, and smart materials, including flexible electronics. They demonstrate immense potential for the leap from a green raw material to a high-value-added functional material.

However, translating SPHs from the laboratory to scalable applications remains a challenge. The key lies in balancing performance, safety, and cost: pure SP hydrogels often lack sufficient mechanical properties, while chemical crosslinking to enhance performance requires careful biosafety evaluation. Understanding the multiscale “structure–property relationship” from molecular conformation to macroscopic performance remains incomplete, leading to performance control that is still somewhat empirical. Efficient, low-cost, continuous large-scale preparation processes are largely absent. Furthermore, a disconnect often persists between material design and the specific requirements of end-use application scenarios.

### 6.2. Future Perspectives

Looking ahead, advancing the field of SPHs hinges on deep interdisciplinary integration and innovation. While the preceding conclusions summarize the current state of the art, the true translational potential of SPHs lies in embracing a multi-tiered roadmap spanning intelligent molecular design, green biomanufacturing, and full-chain industrial translation.

At the primary level of intelligent molecular design, the integration of bioinformatics, machine learning (ML), and computational protein engineering is poised to revolutionize the SPH development cycle. Specifically, ML algorithms can be trained on existing rheological and mechanical datasets to predict the optimal crosslinking density and network topology required for specific applications, thereby dramatically reducing empirical trial-and-error. Furthermore, advanced molecular dynamics (MD) simulations are urgently needed to elucidate the dynamic evolution of 7S/11S aggregation pathways and solvent–protein interactions at the atomic level. Such computational frameworks will enable the rational design of soy protein variants with predetermined gelation kinetics, moving SPH engineering from a “materials-by-chance” to a “materials-by-design” paradigm.

At the secondary level of advanced biomanufacturing, precision fermentation and enzymatic engineering offer unprecedented opportunities to overcome the inherent batch-to-batch variability of agricultural raw materials. By employing engineered microbial cell factories, it is feasible to produce specific soy protein fractions with precisely tuned post-translational modifications or non-canonical amino acid insertions, which can serve as standardized building blocks for high-performance hydrogels. This biological route provides cleaner, more reproducible alternatives to traditional chemical modification, ensuring consistent quality for high-end biomedical and pharmaceutical applications.

At the tertiary level of scalable manufacturing and industrial translation, significant bottlenecks remain in the transition from laboratory-scale batches to continuous industrial production. Future efforts must prioritize the development of continuous extrusion, microfluidic spinning, and high-throughput 3D printing technologies tailored to SPH precursors. Equally crucial is the establishment of standardized characterization protocols to ensure batch-to-batch reproducibility. Concurrently, a full-chain life cycle assessment (LCA) must be systematically conducted to quantitatively benchmark the carbon footprint, water usage, and energy consumption of SPHs against conventional petroleum-based hydrogels and animal-derived proteins, thereby substantiating their ecological advantages. To facilitate regulatory approval and market entry, collaborative research with regulatory bodies is imperative to establish clear safety thresholds for residual crosslinkers, nanoparticles, and degradation byproducts, particularly for food-contact and implantable medical devices. Addressing these translational challenges through robust industry–academia partnerships will be the key to unlocking the commercial viability of SPHs, ensuring they evolve from academic curiosities into mainstream sustainable material solutions.

In summary, as a bridge connecting sustainable resources with cutting-edge functional materials, soy protein hydrogels are at a critical stage, transitioning from rapid knowledge accumulation to breakthroughs in in-depth application. Through sustained interdisciplinary collaboration and innovation, and close industry–academia–research linkages, SPHs hold the promise of delivering more crucial “green” solutions to global challenges in health, food, the environment, and energy, realizing the grand vision from laboratory innovation to industrial transformation.

## Figures and Tables

**Table 2:** Main functional groups in soy protein, corresponding amino acid residues, and their roles in gel crosslinking.

Functional Group Type	Main Residues	Role in Gel Formation	Refs
Amino (-NH_2_)	Lysine (Lys), Arginine (Arg)	Participation in enzymatic crosslinking (TGase), aldehyde crosslinking, EDC/NHS reactions, electrophilic addition, and grafting of photosensitive groups	[9,10,30,31,32]
Carboxyl (-COOH)	Glutamic acid (Glu), Aspartic acid (Asp)	Chelation with multivalent metal ions (Ca^2+^, Fe^3+^), condensation with amino groups, and electrostatic interactions	[9,33,34]
Thiol/Sulfhydryl (-SH)	Cysteine (Cys)	Disulfide bond crosslinking, thiol–ene click reactions	[10,35,36]
Hydroxyl (-OH)	Serine (Ser), Threonine (Thr), Tyrosine (Tyr)	Weak hydrogen bond crosslinking, chemical grafting	[9,11]
Aromatic/Hydrophobic Side Chains	Phenylalanine (Phe), Tyrosine (Tyr), Tryptophan (Trp)	Hydrophobic aggregation, π-π stacking	[10,11,25]

**Table 7:** Summary of Major Application Fields, Strategies, and Key Properties of Soy Protein Hydrogels.

Application Field	Functional Requirement	SPH Design Strategy	Key Properties Achieved	Current Limitations	References
Biomedicine	Biocompatibility, RGD-mediated cell adhesion, Controlled biodegradability, Hemostasis	Composite with inorganics (HAp); IPN with polysaccharides; Functionalization with polyphenols; Drug loading	High porosity, adjustable mechanical strength, antibacterial, antioxidant, pH-responsive release, hemostatic	Immunogenicity of 7S/11S epitopes; Long-term in vivo degradation mismatch; Sterilization process incompatibility	[75,76,77,90,91,92,93,94,95,96,97,98,99,100]
Food and Nutrition	Fibrous texture, Thermal stability, Gastric protection/Intestinal release, Shear-thinning for printing	Composite with polysaccharides; Double-network formation; Emulsion stabilization; 3D printing inks	Anisotropic structure, high water-holding capacity, shear-thinning, probiotic protection, flavor encapsulation	“Beany” off-flavor; High water content limits shelf-life; regulatory acceptance of novel crosslinkers	[78,105,106,107,108,109,110,111,112,113,114,115,116,117]
Environment & Agriculture	High swelling ratio, High adsorption capacity, Biodegradability, Low cost	Composite with biochar, clays, or synthetic polymers; Crosslinking with green reagents	High adsorption capacity, high swelling ratio, slow nutrient release, soil degradation	Trade-off between crosslinking density and adsorption capacity; Complex real-water matrix effects	[79,125,126,127,128,129,130,131,132,133,134,135,136]
Smart Materials	High ionic conductivity, Self-healing/Adhesion, Extreme environmental tolerance, UV-shielding/Antibacterial	Incorporation of conductive fillers (e.g., MXene); Salt-out/Hofmeister effect; Carbonization	High ionic conductivity, stretchability, self-healing, UV-shielding, antibacterial, superhydrophobicity	Long-term stability; Cycle life fatigue; Integration with rigid electronic components; High cost of functional fillers	[80,137,138,139,140,141,142,143,144,145,146,147,148,149,150,151,152,153,154,155,156,157,158]

## Data Availability

All data and materials are available on request from the corresponding author. The data are not publicly available due to ongoing research using a part of the data.

## References

[1] Guo Z., Wang M., Li T., Xiao S., Zhou M., Li Y., Xia C., Chen C., Fu Q., Wang Q. (2026). Tough, Electron-Ion Conductive, and Anti-Freezing Cellulose Nanofiber Reinforced Hydrogels Inspired by Honeycomb as Flexible Wearable Sensors for Intelligent Human Health Monitoring. Chem. Eng. J..

[2] Wang Q., Xue Z., Tang J., Chen M., Wu D., Qin W., Zhang Q. (2026). Crosslinking-Driven Design of Protein Hydrogels: Mechanisms, Engineering Strategies, and Innovative Applications in Food Science. Adv. Colloid Interface Sci..

[3] Kaleshtari A.H., Hasani M., Farjaminejad S., Farjaminejad R., Foroozandeh A., Abdouss M., Hasanzadeh M. (2026). The Critical Role of Hydrogels as an Advanced Polymeric Scaffold in Biomedicine: Recent Progress and Challenges. RSC Adv..

[4] Li S., Yao F., Liu Q., Tang C., Zhuo Y., Dai M., Lv Q., Zhong X. (2025). Natural Polysaccharide Hydrogels: Design, Preparation, and Tissue Engineering Applications. Mater. Des..

[5] Li X., Gong J.P. (2024). Design Principles for Strong and Tough Hydrogels. Nat. Rev. Mater..

[6] Lang Y.F., Dong Y.M., Li X.N., Yan Q., Zeng G.D., Li J.Z. (2026). Recent Progress on the Application of Hyperbranched Polymers in Biomass-Based Adhesives for Sustainable Wood Bonding. ChemSusChem.

[7] Lei Z., Shao J., Li C., Jiang S., Yao M., Li J. (2025). Skin-Inspired Durable and Cost-Effective Biomass-Based Supramolecular Adhesives. Adv. Funct. Mater..

[8] Karim A., Osse E.F., Khalloufi S. (2025). Innovative Strategies for Valorization of Byproducts from Soybean Industry: A Review on Status, Challenges, and Sustainable Approaches Towards Zero-Waste Processing Systems. Heliyon.

[9] Cho E.-S., Kim S., Moon J.-K., Park S.-K., Maruyama N., Wang S., Lee C.-H., Lee J.-Y. (2025). Identification and Quantification of Soybean 11s and 7s Globulins Using Rp-Uplc. Food Chem..

[10] Kang S., Bai Q., Qin Y., Liang Q., Hu Y., Li S., Luan G. (2023). Film-Forming Properties and Mechanisms of Soy Protein: Insights from Β-Conglycinin and Glycinin. Int. J. Biol. Macromol..

[11] Din J.U., Sarwar A., Li Y., Aziz T., Hussain F., Shah S.M.M., Yi G., Liu X. (2021). Separation of Storage Proteins (7s and 11s) from Soybean Seed, Meals and Protein Isolate Using an Optimized Method Via Comparison of Yield and Purity. Protein J..

[12] Joyce K., Fabra G.T., Bozkurt Y., Pandit A. (2021). Correction To: Bioactive Potential of Natural Biomaterials: Identification, Retention and Assessment of Biological Properties. Signal Transduct. Target. Ther..

[13] Singhal P., Vashisht H., Nisar S., Mehra S., Rattan S. (2022). Stimulus Responsive Soy-Protein Based Hydrogels through Grafting Hema for Biomedical Applications. Ind. Crops Prod..

[14] Liu Y., Dong F., Zhou L., Zhao Q., Zhang S. (2025). Development of Soybean Protein-Based Bioactive Substances Delivery Systems: A Systematic Overview Based on Recent Researches. Int. J. Biol. Macromol..

[15] Zhang T., Zhang R., Zhao C., Li Z., Wang L., Zhao H. (2025). Preparation, Characterization, Multidimensional Applications and Prospects of Protein Bio-Based Hydrogels: A Review. Int. J. Biol. Macromol..

[16] Cairang Z., Shi C., Ou H., Wu Y., Fang D., Li W. (2026). Structure-Function Relationship and Intelligent Regulation of Polysaccharide and Protein-Based Hydrogels in Food Fresh-Keeping Packaging: A Review. Int. J. Biol. Macromol..

[17] Arif M., Yang Q., Kanwal A., Zhao J., Nawaz M., Yang P., Ren H. (2025). Sustainable Rhenium Adsorption and Separation from Molybdenum-Containing Solution Using Soy Protein-Based Material. J. Environ. Chem. Eng..

[18] Tang Q., Roos Y.H., Miao S. (2024). Structure, Gelation Mechanism of Plant Proteins Versus Dairy Proteins and Evolving Modification Strategies. Trends Food Sci. Technol..

[19] Guo J., Xie Q., Sun Y., Guo W., Liu Y., Jiang Y., Guo L., Zhang Y. (2026). Enzymatic Cross-Linking Chemical Modification and Ultrasound to Improve the Structural and Functional Properties of Soy Protein Isolate. Food Res. Int..

[20] Zhu Y., Wu H., Yang W., Charrier B., Essawy H., Pizzi A., Zhou X., Chen X. (2026). Modification of Nanocellulose Filler Via Amination and Subsequent Grafting of Gallic Acid for Enhancing the Performance of Biodegradable Soy Protein Films in Banana Packaging. Food Chem. X.

[21] Hou X., Sun F., Liu X., Tu W., Wu K., Zhao G., Zhang B., Qiao D. (2025). Micro-Phase Separation Dual-Network Strategy Enhances the Gel Structure and Gel-Related Properties of Soy Bean Protein Heat-Induced Gel by Incorporation of Curdlan. Food Chem..

[22] Lei D., Li J., Zhang C., Li S., Zhu Z., Wang F., Deng Q., Grimi N. (2022). Complexation of Soybean Protein Isolate with Β-Glucan and Myricetin: Different Affinity on 7s and 11s Globulin by Qcm-D and Molecular Simulation Analysis. Food Chem. X.

[23] Sun P., Zhang Q., Zhao Y., Zhao D., Zhao X., Jiang L., Zhang Y., Wu F., Sui X. (2023). Improving Gel Properties of Soy Protein Isolate through Alkaline Ph-Shifting, Mild Heat Treatment, and Tgase Cross-Linking. Food Hydrocoll..

[24] Jegadeesan S., Yu K., Woodrow L., Wang Y., Shi C., Poysa V. (2012). Molecular Analysis of Glycinin Genes in Soybean Mutants for Development of Gene-Specific Markers. Theor. Appl. Genet..

[25] Guo J., Yang X.-Q., He X.-T., Wu N.-N., Wang J.-M., Gu W., Zhang Y.-Y. (2012). Limited Aggregation Behavior of Β-Conglycinin and Its Terminating Effect on Glycinin Aggregation During Heating at Ph 7.0. J. Agric. Food Chem..

[26] Hsiao Y.-H., Yu C.-J., Li W.-T., Hsieh J.-F. (2015). Coagulation of Β-Conglycinin, Glycinin and Isoflavones Induced by Calcium Chloride in Soymilk. Sci. Rep..

[27] Holzhauser T., Wackermann O., Ballmer-Weber B.K., Bindslev-Jensen C., Scibilia J., Perono-Garoffo L., Utsumi S., Poulsen L.K., Vieths S. (2009). Soybean (*Glycine max*) Allergy in Europe: Gly M 5 (Β-Conglycinin) and Gly M 6 (Glycinin) Are Potential Diagnostic Markers for Severe Allergic Reactions to Soy. J. Allergy Clin. Immunol..

[28] Ma H., Li J., Guan Y., Song Z., Chen H., Sun S. (2025). Structural and Functional Properties of Soy Protein Isolates from Different Cultivars. Int. J. Biol. Macromol..

[29] Qin P., Wang T., Luo Y. (2022). A Review on Plant-Based Proteins from Soybean: Health Benefits and Soy Product Development. J. Agric. Food Res..

[30] Cheng X., Wang Z., Guo Z., Xu J. (2026). Oxidation-Engineered in Soy Protein-Oxidized Alginate in Situ Hydrogels: The Evolution from “Point-Point” to “Line-Line” Via Dynamic Schiff Base Crosslinking. Food Res. Int..

[31] Xu J., Wang Q., Yan S., Qi B. (2025). Hydrogel Formed in Situ through Schiff Base Reaction between Gallic Acid-Grafted Soybean Protein Isolate and Oxidized Dextran: Interactions, Physicochemical Properties, and Digestive Characteristics. Food Chem..

[32] Liu J., Li Z., Lin Q., Jiang X., Yao J., Yang Y., Shao Z., Chen X. (2018). A Robust, Resilient, and Multi-Functional Soy Protein-Based Hydrogel. ACS Sustain. Chem. Eng..

[33] Li T.-H., Yang Y.-Q., Wang G.-S., Lv D.-Y., Guo J., Wan Z.-L., Yang X.-Q. (2025). Effects of Salt Ions and Ph on Deamidated Soybean Protein Hydrogels Formation: Molecular Structure, Thermal Aggregation and Network. Food Chem..

[34] Wang Q., Chen T., Xu J., Yan S., Qi B. (2025). Soybean Protein Isolate/Sodium Alginate Double-Network Hydrogels with High Mechanical Strength Via Synergistic Cross-Linking of Imine and Ionic Bonds. Food Chem..

[35] Ji S., Ma N., Zhang X., Ding S., Yan X., Wang X., Xu N. (2026). Mechanistic Insights into Sericin-Soy Protein Isolate Co-Gelation: Molecular Interactions, Interfacial Functionality and Gel Network Reinforcement. Food Hydrocoll..

[36] Li T.-H., Yang Y.-Q., Lv D.-Y., Wang G.-S., Guo J., Wan Z.-L., Yang X.-Q. (2024). Formation of a Transparent Soy Protein Hydrogel: Controlled Thermal Aggregation of Protein Using Glutaminase. Food Hydrocoll..

[37] Wang L., Dong Y., Wang L., Cui M., Zhang Y., Jiang L., Sui X. (2023). Elucidating the Effect of the Hofmeister Effect on Formation and Rheological Properties of Soy Protein/Κ-Carrageenan Hydrogels. Food Hydrocoll..

[38] Cheng X., Hu T., Su H., Xie Y., Yuan Y., Hu H. (2026). Short-Time High-Intensity Ultrasound Directly Induces Soy Protein Isolate-Glucono-Δ-Lactone Gels. Food Hydrocoll..

[39] Chen L., Lu H., Qu L., Zhao Y., He R., Huang L., Zhang R. (2026). Impact of Sonication and Sodium Chloride on the Interaction and Gelation Behavior of Soy Protein Isolate/Κ-Carrageenan Mixtures. Food Chem..

[40] Zhu Y., Wang Y., Zhang L., Chen X., Dong Y., Zou L., Liu W. (2025). Engineering Soy Protein/Konjac Glucomannan Double Network Hydrogels with Low Voltage-Electric Field: Anisotropic Structure and Properties. Food Res. Int..

[41] Wang Y., Wang N., Yang H., Wang T., Han C., Yu D. (2026). Development of a Novel Soybean Protein Isolate—Rice Bran Wax Bigel System: Effects of Alternating Electric Field Pretreatment and Hydrogel/Oleogel Ratio on Bigel Properties. Food Hydrocoll..

[42] Florowska A., Florowski T., Goździk P., Hilal A., Florowska H., Janiszewska-Turak E. (2024). The Effect of High Hydrostatic Pressure (HHP) Induction Parameters on the Formation and Properties of Inulin–Soy Protein Hydrogels. Gels.

[43] Guo Y., Zhang Z.-G., Cai J., Li W.-R., Chen L.-Y., Wu W.-C. (2023). Corrigendum to “Co-Folding of Soy Protein Isolates and Shellac by Structural Interplays to Induce Hydrogels” [Food Hydrocolloids 139 (2023) 108527]. Food Hydrocoll..

[44] Wang Q., Chen T., Zhu Y., Ning Y., Li Y., Yan S., Qi B. (2025). Soybean Protein Isolate/Dialdehyde Sodium Alginate Hydrogels Based on Dynamic Imine Bond Cross-Linking: Synthesis and Properties. Food Res. Int..

[45] Liu J., Zhou H., Huang Y.F., Chen X. (2018). Polyethylene Glycol Chemically Modified Soy Protein Isolate Hydrogel. Chem. J. Chin. Univ..

[46] Song W., Xin J., Zhang J. (2017). One-Pot Synthesis of Soy Protein (Sp)-Poly(Acrylic Acid) (Paa) Superabsorbent Hydrogels Via Facile Preparation of Sp Macromonomer. Ind. Crops Prod..

[47] Li L., Liu Y., Wu X., Geng M., Teng F., Li Y. (2024). Interpenetrating Network Based on Soybean Protein Isolate and Carboxymethyl Chitosan: Investigation of Intermolecular Reaction Mechanism, Physicochemical Stability and in Vitro Release Properties of the Hydrogel. Food Biosci..

[48] Yan S., Wang Q., Li Y., Qi B. (2024). Gallic Acid-Functionalized Soy Protein-Based Multiple Cross-Linked Hydrogel: Mechanism Analysis, Physicochemical Properties, and Digestive Characteristics. Food Chem..

[49] Shi J., Lan T., Wang B., Dong Y., Xu Z., Zhang Y., Sui X. (2025). Influence of Metal-Phenolic Networks on the Properties of Soy Protein Isolates Gels. Food Hydrocoll..

[50] Ding S., Ma N., Zhang M., Ji S., Jia B., Nan D., Wang X., Xu N. (2026). Tailoring the Structure and Function of Soy Protein Isolate Hydrogels Via Cinnamaldehyde Cross-Linking: From Molecular Interactions to Gel Characteristics. Food Res. Int..

[51] Peng X., Liu Y., Chi Q., Li J., Dai S., Tong X., Wang H., Jiang L. (2025). Phased Characterization of Soy Protein Gel Modified by *Lactobacillus plantarum* Jylp-326 in Cooperation with Acidic Tremella Fuciformis Fruiting Body Polysaccharide: Focus on Structural Network, Interaction and Gel Properties. Food Chem..

[52] Jin M., Ikeda S., Zhong Q. (2013). Strengthening Soy Protein Hydrogels Filled with Protein-Coated Montmorillonite Nanoclay by Glutaraldehyde Crosslinking. Food Sci. Technol..

[53] Yang S., Chi Y., Guo X., Zhang J., Lian Z., Tong X., Wang H., Jiang L. (2025). Carboxylated Cellulose Nanocrystals-Driven Heat-Induced Soy Protein Isolate Amyloid Fibril Gel: Network Structure and Formation Mechanism. Food Chem..

[54] Liang G., Chen W., Wang Z., Chu X., Zeng M., He Z., Goff H.D., Chen J. (2025). Hydrogel Structure of Soy Protein and Gelatin Dual Network Based on Tgase Cross-Linking. Food Hydrocoll..

[55] Gan J., Sun L., Guan C., Ren T., Zhang Q., Pan S., Zhang Q., Chen H. (2022). Preparation and Properties of Salecan–Soy Protein Isolate Composite Hydrogel Induced by Thermal Treatment and Transglutaminase. Int. J. Mol. Sci..

[56] Wang L., Dong Y., Jiang L., Zhang Y., Sui X. (2025). The Hofmeister Series: Anion Effect on Microbial Transglutaminase Cross-Linked Soybean Protein Isolate Hydrogels. Food Chem..

[57] Zhao R., Wu L., Gao Y., Wang C.Y., Bai X.H., Luo S.Z., Zheng Z. (2024). Fabrication and Characterization of Soy Protein Isolation-Ferulic Acid Antioxidant Hydrogels. J. Sci. Food Agric..

[58] Guo J., Zhou Q., Liu Y.-C., Yang X.-Q., Wang J.-M., Yin S.-W., Qi J.-R. (2016). Preparation of Soy Protein-Based Microgel Particles Using a Hydrogel Homogenizing Strategy and Their Interfacial Properties. Food Hydrocoll..

[59] Li Y., Wang S., Liu X., Yang L., Liu H., He Y. (2023). Characterization of Enzymatic Cross-Linking Soy Protein Isolate Xerogels and Its Shape Memory Effect Induced by Ethylcellulose. Food Chem..

[60] Lu Y., Guo Y., Jin J., Xie J., Dong X., Qi H. (2026). Preparation of Ph-Responsive Hydrogel Microspheres Based on Fibrillated Soy Protein Isolate and Sodium Alginate and Investigation of Polyphenol Release Behavior from Ascophyllum Nodosum. Food Hydrocoll..

[61] Yan W., Jia X., Zhang Q., Chen H., Zhu Q., Yin L. (2021). Interpenetrating Polymer Network Hydrogels of Soy Protein Isolate and Sugar Beet Pectin as a Potential Carrier for Probiotics. Food Hydrocoll..

[62] Qi W., Zhang Y., Li S., Nie M., Jiang L., Sun Y., Sui X. (2026). Polysaccharide Structure–Activity Mechanisms Regulating Spi-Pp Composite Hydrogels. Food Chem..

[63] Luo Y., Wang Y., Li X., Zhang Y., Wang J., Xu Z., Sui X. (2026). Compact Hydrogels Network Structures Reinforced by Soy Protein Amyloid Fibrils and Sodium Alginate. Food Res. Int..

[64] Zhao Y., Shan Y., Wang H., Zhang Z., Wang C., Dai L., Wang Y., Sun Q., McClements D.J., Cheng Y. (2026). Interpenetrating Network Hydrogels Based on Soy Protein Isolate and Sanxan: Structure, Mechanical Properties, and Freeze-Thaw Stability. Carbohydr. Polym..

[65] Khan A., Hu T., Chen W., Rahman Z., Khan S.A., Yuan Y., Hu H. (2026). Mechanistic Insights into Soy Protein Isolate Gelation through Polyphenol-Cellulose Pre-Complex Strategy: Gelation Behavior and Structural Properties. Food Res. Int..

[66] Xu J., Yan S., Qi B., Jiang L. (2025). New Insights into the Cross-Linking Mechanism of Soybean Protein-Based Double Dynamic Cross-Linking Hydrogels for the Controlled Delivery of Curcumin. Food Res. Int..

[67] Chen Q.-H., Li X.-Y., Huang C.-L., Liu P., Zeng Q.-Z., Yang X.-Q., Yuan Y. (2021). Development and Mechanical Properties of Soy Protein Isolate-Chitin Nanofibers Complex Gel: The Role of High-Pressure Homogenization. LWT.

[68] Dai N., Xia J., Dai Z., Chen J., Li C., Geng Q., Deng L., Li T., Dai T. (2026). Enhancement by Gellan Gum on Acid-Induced Gels of Rice-Soy Protein Using High-Energy Fluidic Microfluidizer Coupled with Alkali-Driven Pretreatment. J. Future Foods.

[69] Fu Y., Tian R., Zhao J., Wang J., Sui X. (2025). Elucidation of the Thermal Aggregation Processing of Soy Protein Isolate—Dialdehyde Carboxymethyl Cellulose Cross-Linked Gel. Food Chem..

[70] Ma C., Pang H., Liu H., Yan Q., Li J., Zhang S. (2021). A Tough, Adhesive, Self-Healable, and Antibacterial Plant-Inspired Hydrogel Based on Pyrogallol–Borax Dynamic Cross-Linking. J. Mater. Chem. B.

[71] Chang Z., Shen Y., Xue J., Sun Y., Zhang S. (2023). Multifunctional Soy Protein Gels with Excellent Initial Adhesion and Bonding Strength Based on a Mussel-Inspired Redox Self-Catalytic and Oyster-Inspired Organic-Inorganic Hybrid Dual-Bionic Strategy. Ind. Crops Prod..

[72] Sun W., Bu K., Meng H., Zhu C. (2024). Hawthorn Pectin/Soybean Isolate Protein Hydrogel Bead as a Promising Ferrous Ion-Embedded Delivery System. Colloids Surf. B.

[73] Zhao Y.T., Jin L., Liu X., Liu X., Dong S.L., Chen Y., Li X.Y., Lv X.P., He M. (2022). Novel High Strength Pva/Soy Protein Isolate Composite Hydrogels and Their Properties. Front. Chem..

[74] Yan J., Jia X., Yan W., Yin L. (2021). Double-Network Hydrogels of Corn Fiber Gum and Soy Protein Isolate: Effect of Biopolymer Constituents and pH Values on Textural Properties and Microstructures. Foods.

[75] Liu Y., Hu Q., Dong W., Liu S., Zhang H., Gu Y. (2022). Alginate/Gelatin-Based Hydrogel with Soy Protein/Peptide Powder for 3d Printing Tissue-Engineering Scaffolds to Promote Angiogenesis. Macromol. Biosci..

[76] Huang X., Ma C., Xu Y., Cao J., Li J., Li J., Shi S.Q., Gao Q. (2022). A Tannin-Functionalized Soy Protein-Based Adhesive Hydrogel as a Wound Dressing. Ind. Crops Prod..

[77] Tao R., Li G., Wang S., Sun Y., Li Y., Wang P., Huang S., Fan B., Wang F. (2025). Natural, Tough and Ph-Responsive Ultrasound Modified Soy Protein Isolate-Arabinoxylan Based Double Network Hydrogels for Controlled Nutrients Release. Food Hydrocoll..

[78] Xia P., Miyajima H., Fujita S. (2025). Development of Biomimetic Edible Scaffolds for Cultured Meat Based on the Traditional Freeze-Drying Method for *Ito-Kanten* (Japanese Freeze-Dried Agar). Gels.

[79] Liang J., Zhao Z., Xing M., Wang X., Dong Y., Yang Y., Du N., Gu H., Meng L., Peng W. (2024). Improving Properties of Soy Protein–Based Hydrogel Composites by Incorporating Bamboo Biochar Towards Slow Release and Water Retention of Fertilizers and Enhanced Plant Growth. Adv. Compos. Hybrid Mater..

[80] Deng Y., Yang M., Xiao G., Jiang X. (2024). Preparation of Strong, Tough and Conductive Soy Protein Isolate/Poly(Vinyl Alcohol)-Based Hydrogel Via the Synergy of Biomineralization and Salting Out. Int. J. Biol. Macromol..

[81] Zou B., Zheng X., Sun R., Na X., Du M., Wu C. (2025). Accelerated Gelation and Strengthened Network Formation in Whey-Pea Binary Protein Systems Via Linear or Branched Β-Glucans. Carbohydr. Polym..

[82] Seelig J., Seelig A. (2023). Protein Unfolding—Thermodynamic Perspectives and Unfolding Models. Int. J. Mol. Sci..

[83] Momen S., Sawant S., Lincoln L., Fallen B.D., Girard A.L. (2026). Impact of Soybean Genotype Diversity on the Structure and Gelation Properties of Soy Proteins. Food Chem. Mol. Sci..

[84] Fu H., Li J., Yang X., Swallah M.S., Gong H., Ji L., Meng X., Lyu B., Yu H. (2023). The Heated-Induced Gelation of Soy Protein Isolate at Subunit Level: Exploring the Impacts of A and A′ Subunits on Spi Gelation Based on Natural Hybrid Breeding Varieties. Food Hydrocoll..

[85] Kopač T., Abrami M., Grassi M., Ručigaj A., Krajnc M. (2021). Polysaccharide-Based Hydrogels Crosslink Density Equation: A Rheological and Lf-Nmr Study of Polymer-Polymer Interactions. Carbohydr. Polym..

[86] Cao H., Duan L., Zhang Y., Cao J., Zhang K. (2021). Current Hydrogel Advances in Physicochemical and Biological Response-Driven Biomedical Application Diversity. Signal Transduct. Target. Ther..

[87] Tolentino M.A.K., Du E.Y., Silvani G., Pandzic E., Kilian K.A., Gooding J.J. (2025). Decoding Hydrogel Porosity: Advancing the Structural Analysis of Hydrogels for Biomedical Applications. Adv. Healthc. Mater..

[88] Wang Y., Zhang S., Zheng L. (2025). Soy Protein-Based Protein Composite System: Gelation, Application, and Challenges—A Review. Eur. Food Res. Technol..

[89] Fan Z., Cheng P., Zhang P., Zhang G., Han J. (2022). Rheological Insight of Polysaccharide/Protein Based Hydrogels in Recent Food and Biomedical Fields: A Review. Int. J. Biol. Macromol..

[90] Alesaeidi S., Kahrizi M.S., Ghorbani Tajani A., Hajipour H., Ghorbani M. (2023). Soy Protein Isolate/Sodium Alginate Hybrid Hydrogel Embedded with Hydroxyapatite for Tissue Engineering. J. Polym. Environ..

[91] Mohamad Zulkifli N.A.S., Ng K., Ang B.C., Muhamad F. (2025). Fabrication of Water-Stable Soy Protein Isolate (Spi)/Carboxymethyl Cellulose (Cmc) Scaffold Sourced from Oil Palm Empty Fruit Bunch (Opefb) for Bone Tissue Engineering. Ind. Crops Prod..

[92] Li J., Li Y., Guo C., Wu X. (2024). Development of Quercetin Loaded Silk Fibroin/Soybean Protein Isolate Hydrogels for Burn Wound Healing. Chem. Eng. J..

[93] Varshney N., Singh P., Rai R., Vishwakarma N.K., Mahto S.K. (2023). Superporous Soy Protein Isolate Matrices as Superabsorbent Dressings for Successful Management of Highly Exuding Wounds: In Vitro and in Vivo Characterization. Int. J. Biol. Macromol..

[94] Yanan Z., Ao X., Ping W., Feixiang C., Qiang Z., Xiao L., Xinwei H., Xiaowen S., Yinping L., Yun C. (2021). Fabrication of Hydroxypropyl Chitosan/Soy Protein Isolate Hydrogel for Effective Hemorrhage Control. Tissue Eng. Part A.

[95] Zhou D., Liu H., Han L., Liu D., Liu X., Yan Q., He D., Li Z., Lu X., Jiang C. (2023). Paintable Graphene Oxide-Hybridized Soy Protein-Based Biogels for Skin Radioprotection. Chem. Eng. J..

[96] He L., Li G., Wang H., Li Y., Ma Y., Cui J., Shen J., Zhang J., Liu Y., Ma X. (2024). Rapidly Molded Sodium Alginate/Soy Protein Adhesive Hydrogel with 808-Nm Laser Inhibition of Infected Wounds. Int. J. Biol. Macromol..

[97] Liu H., Lu X. (2026). Phototriggered Self-Adhesive Soybean Protein-Based Hydrogels for Promoting Chronic Wound Healing. ACS Appl. Polym. Mater..

[98] Yan J., Yang H., Cao S., Pang M., Yao G., Qi X., Mao B. (2025). Dual-Induced Sorghum Bran Arabinoxylan and Soy Protein Isolate Composite Gels: Insights into Their Formation and Application as Bioactive Component Delivery Carriers. LWT.

[99] Cui Q., Song X., Zhou L., Dong J., Wei Y., Liu Z., Wu X. (2025). Corrigendum to “Fabrication of Resveratrol-Loaded Soy Protein Isolate-Glycyrrhizin Nanocomplex for Improving Bioavailability Via Ph-Responsive Hydrogel Properties” [Int. J. Biol. Macromol. 2024 Feb; 258(Pt 1):128950]. Int. J. Biol. Macromol..

[100] Viale F., Ciprandi M., Leoni L., Sierri G., Renda A., Barbugian F., Koch M., Sesana S., Salvioni L., Colombo M. (2024). Biodegradable Spi-Based Hydrogel for Controlled Release of Nanomedicines: A Potential Approach against Brain Tumors Recurrence. ACS Appl. Polym. Mater..

[101] Argenziano R., Viggiano S., Esposito R., Schibeci M., Gaglione R., Castaldo R., Fusaro L., Boccafoschi F., Arciello A., Della Greca M. (2023). All Natural Mussel-Inspired Bioadhesives from Soy Proteins and Plant Derived Polyphenols with Marked Water-Resistance and Favourable Antibacterial Profile for Wound Treatment Applications. J. Colloid Interface Sci..

[102] Yang H., Qu Y., Li J., Liu X., Wu R., Wu J. (2020). Improvement of the Protein Quality and Degradation of Allergens in Soybean Meal by Combination Fermentation and Enzymatic Hydrolysis. LWT.

[103] Pi X., Sun Y., Fu G., Wu Z., Cheng J. (2021). Effect of Processing on Soybean Allergens and Their Allergenicity. Trends Food Sci. Technol..

[104] Isaac A.H., Recalde Phillips S.Y., Ruben E., Estes M., Rajavel V., Baig T., Paleti C., Landsgaard K., Lee R.H., Guda T. (2024). Impact of Peg Sensitization on the Efficacy of Peg Hydrogel-Mediated Tissue Engineering. Nat. Commun..

[105] Yang Z., Song K., Wan Z., Guo J., Yang X. (2026). Engineering Anisotropic Hydrogels Via Directional Freezing and Salting-out for Plant-Based Meat Analogues. Food Hydrocoll..

[106] Liu S.Q., Zhao D., Sun L.Y., Ye X.N., Cao J.N., Li H., Liu X.Q. (2024). Investigation into the Fabrication of Plant-Based Simulant Connective Tissue Utilizing Algae Polysaccharide-Derived Hydrogel. Int. J. Biol. Macromol..

[107] Nie C.-Z., Wang J., Tian H.-H., Huang X.-H., Zhu B.-W., Qin L. (2026). Enzyme- and Ion-Induced High Internal Phase Emulsion Gels Based on Soy Protein Isolate, Chitosan, and Alginate as Fat Analogues: Tunable Texture and Controlled Flavor Release. Int. J. Biol. Macromol..

[108] Kim Y.H., Lee S.J., Kil B.J., Chang Y.H. (2026). Preparation of Multipolymer Hydrogel System-Based Boiled Fish Analog for Mimicking Fish Muscle Fiber: Combination of Heat and Non-Heat Matrixes. Food Hydrocoll..

[109] Zhang K., Lei H., Zhang T., Xu J., Liu Z., Yin F., Zhou D., Li D. (2026). Soy Protein Isolate-Konjac Glucomannan Hydrogel as a Sodium Reservoir for Flavor Modulation and Texture Enhancement in Low-Salt Antarctic Krill Surimi. Food Res. Int..

[110] Pan H., Pei F., Ma G., Ma N., Zhong L., Zhao L., Hu Q. (2022). 3d Printing Properties of *Flammulina velutipes* Polysaccharide-Soy Protein Complex Hydrogels. J. Food Eng..

[111] Liu Z., Ruan M., Yin X., Liang W., Xu B., Li H., Yao L., Tian L., Zhang J., Mo H. (2026). Tailoring 3d-Printed Soy Protein Gels for Dysphagia Diets: Influence of Xanthan Gum’s Substituents and Underlying Mechanism. Int. J. Biol. Macromol..

[112] Patil T.D., Gaikwad A., Gaikwad K.K. (2026). Citric Acid-Crosslinked Guar Gum–Carboxymethyl Cellulose–Soy Protein Hydrogel Networks Based on Barnyard Millet for Functional and Sustainable Edible Straws. Int. J. Biol. Macromol..

[113] Praepanitchai O.A., Noomhorm A., Anal A.K. (2019). Survival and Behavior of Encapsulated Probiotics (*Lactobacillus plantarum*) in Calcium-Alginate-Soy Protein Isolate-Based Hydrogel Beads in Different Processing Conditions (Ph and Temperature) and in Pasteurized Mango Juice. BioMed Res. Int..

[114] Virk M.S., Virk M.A., Liang Q., Sun Y., Zhong M., Tufail T., Rashid A., Qayum A., Rehman A., Ekumah J.-N. (2024). Enhancing Storage and Gastroprotective Viability of *Lactiplantibacillus plantarum* Encapsulated by Sodium Caseinate-Inulin-Soy Protein Isolates Composites Carried within Carboxymethyl Cellulose Hydrogel. Food Res. Int..

[115] Liu X., Wang S., Lu G., Han L., Liu H. (2026). Dual-Phase Bigels from Soy Hull Polysaccharide-Soy Protein Isolate Hydrogel and Soy Lecithin-Stearic Acid Oleogel: A Novel Strategy for Probiotic Encapsulation and Gastrointestinal Stability. Food Chem..

[116] Yan W., Zhang B., Yadav M.P., Feng L., Yan J., Jia X., Yin L. (2020). Corn Fiber Gum-Soybean Protein Isolate Double Network Hydrogel as Oral Delivery Vehicles for Thermosensitive Bioactive Compounds. Food Hydrocoll..

[117] Zhou Y., Chai Y., Xu S., Shao P. (2026). Dual Network Pectin-Soy Protein Isolate Gels Loaded with Composite Resveratrol Particles for Ready-to-Drink Innovation. J. Future Foods.

[118] Xu J., Zeng M., Wang Z., Chen Q., Qin F., Chen J., He Z. (2025). Characteristic Flavor Compounds in Soy Protein Isolate and Their Correlation with Sensory Attributes. Food Res. Int..

[119] Wang B., Zhang Q., Zhang N., Bak K.H., Soladoye O.P., Aluko R.E., Fu Y., Zhang Y. (2021). Insights into Formation, Detection and Removal of the Beany Flavor in Soybean Protein. Trends Food Sci. Technol..

[120] Huang J., Vardhanabhuti B. (2026). Off-Flavor Challenges and Mitigation Strategies of Soy Protein Products. Food Biosci..

[121] Abotsi E.E., Panagodage Y., English M. (2024). Plant-Based Seafood Alternatives: Current Insights on the Nutrition, Protein-Flavour Interactions, and the Processing of These Foods. Curr. Res. Food Sci..

[122] Liu Y., Zhang X., Du Y., Du X., Zhang Y., Deng L., Li C., Guo J. (2025). Functionalization of Wood for the Removal of Heavy Metal Ions from Waster Water: A Review. Forests.

[123] Li C., Zhang X., Zhou C., Yang F., Liang J., Gu H., Wang J., Wang F., Peng W., Guo J. (2024). Performance and Mechanism of a Novel Bamboo-Based Magnetic Biochar Composite for Efficient Removal of Norfloxacin. Adv. Compos. Hybrid Mater..

[124] Li H.Y., Zhou C., Wang L.H., Yang F., Liang J.Y., Wang F., Li P.Y., Li C., Wu Z.G., Ren T.B. (2024). A Novel Eco-Friendly Bamboo-Based Composite Biochar for Effective Removing Oxytetracycline Hydrochloride. Adv. Compos. Hybrid Mater..

[125] Liu J., Su D., Yao J., Huang Y., Shao Z., Chen X. (2017). Soy Protein-Based Polyethylenimine Hydrogel and Its High Selectivity for Copper Ion Removal in Wastewater Treatment. J. Mater. Chem. A.

[126] Cao M., Peng Q., Wang Y., Luo G., Feng L., Zhao S., Yuan Y., Wang N. (2023). High-Efficiency Uranium Extraction from Seawater by Low-Cost Natural Protein Hydrogel. Int. J. Biol. Macromol..

[127] Cao Y., Huang M., Zhang Z., Wang Y., He Y.-C. (2024). Antibacterial and Adsorption Properties of Silver-Loaded Carbon-Sodium Alginate-Chitosan-Soy Protein Isolate Composite Gel. Ind. Crops Prod..

[128] Liu F., Yang Q., Tang Q., Peng Q., Chen Y., Huo Y., Huang Q., Zuo Q., Gao N., Chen L. (2023). Adsorption of Rhb Dye on Soy Protein Isolate-Based Double Network Spheres: Compromise between the Removal Efficiency and the Mechanical Strength. Chem. Eng. Res. Des..

[129] Ardra Ashok K.P., Saeed P.A., Smitha M., Sujith A. (2024). Polyvinyl Alcohol-Soy Protein Isolate Hydrogels: Controlled Release of Fertilizer and Matrix Nutrients for Sustainable Agriculture. J. Clean. Prod..

[130] Jiménez-Rosado M., Perez-Puyana V., Rubio-Valle J.F., Guerrero A., Romero A. (2020). Evaluation of Superabsorbent Capacity of Soy Protein-Based Bioplastic Matrices with Incorporated Fertilizer for Crops. J. Polym. Environ..

[131] Jiménez-Rosado M., Martín A., Alonso-González M., Guerrero A., Romero A. (2020). Functional Biodegradable Protein-Based Matrices as a Potential Candidate for Micronutrients and Water Supply. Polym. Eng. Sci..

[132] Jiménez-Rosado M., Perez-Puyana V., Guerrero A., Romero A. (2021). Controlled Release of Zinc from Soy Protein-Based Matrices to Plants. Agronomy.

[133] Wang L., Ji X., Cheng Y., Tao Y., Lu J., Du J., Wang H. (2022). All-Biodegradable Soy Protein Isolate/Lignin Composite Cross-Linked by Oxidized Sucrose as Agricultural Mulch Films for Green Farming. Int. J. Biol. Macromol..

[134] Zhang B., Song H., Zhang Y., Wei H., Liu Y., Zhang Q., Wang G., Guo T., Yan M., Yang X. (2026). Facile Preparation of Ph-/Temperature-/Ion-Sensitive Sensitive Protein/Starch Hydrogel Microparticles Incorporating Zinc-Based Metal-Organic Frameworks by Reactive Spray Drying for Ciprofloxacin Removal. Colloids Surf. A.

[135] Cuadri A.A., Romero A., Bengoechea C., Guerrero A. (2017). Natural Superabsorbent Plastic Materials Based on a Functionalized Soy Protein. Polym. Test..

[136] Ma Y., Dong Q. (2023). Soybean Protein Isolate-Incorporated Zwitterionic Hydrogel with Rapid Chlorine Rechargeable Biocidal and Antifouling Functions. ACS Sustain. Chem. Eng..

[137] Wu Z., Wang L., Ma C., Xu M., Guan X., Lin F., Jiang T., Chen X., Bu N., Duan J. (2025). Konjac Glucomannan/Xanthan Gum Film Embedding Soy Protein Isolate–Tannic Acid–Iron Complexes for Blueberry Preservation. Food Hydrocoll..

[138] Cheng H., McClements D.J., Xu H., Zhu H., Zhang R., Zhang Z., Jin Z., Chen L. (2026). Corn Starch-Soy Protein Isolate Films Reinforced by Metal-Phenolic Networks: Properties and Application for Strawberry Preservation. Food Chem..

[139] Sun Y., Mou K., Bai L., Cheng S., Su W., Wang H., Tan M. (2026). Goose Feather-Inspired Soy Protein Isolate Films Incorporating L-Cys@Cu Nanofibers for Sustainable Packaging. Chem. Eng. J..

[140] Shi C., Yang R., Zhao Y., Cai R., Aziz T., Shami A., Al-Asmari F., Al-Joufi F.A., Cui H., Lin L. (2026). Enhancement of Blueberry Preservation with an Antifungal and Cushioning Soy Protein Isolate Porous Hydrogels Incorporated with Litsea Cubeba Essential Oil Nanoemulsions. Int. J. Biol. Macromol..

[141] Meng D., Yang W., Zhang Z., Wang X., Li J., Yuan L. (2026). Soy Protein Isolate-Pectin from *Nicandra Physaloides* (Linn.) Gaertn Seeds Nanogels: A Hydrocolloid System for Blueberry Preservation. Food Chem. X.

[142] Wang L., Li D., Xue Y.T., Li S.Y., Yang X.X., Li L., Li T.F., Luo Z.S. (2023). Fabrication and Characterization of Novel Porous Hydrogels for Fragile Fruits: A Case Study. Food Hydrocoll..

[143] Li H., Liu M., Li J., Zhang X., Zhang H., Zheng L., Xia N., Wei A., Hua S. (2024). 3d Printing of Smart Labels with Curcumin-Loaded Soy Protein Isolate. Int. J. Biol. Macromol..

[144] Lv Y., Xu Y., Yang W., Sun C., Li G., Fang Z., Qiao Z. (2025). Application of Conductive Hydrogel Based on Polyacrylamide/Maleic Anhydride Modified Soy Protein Isolate in Flexible Sensor. Eur. Polym. J..

[145] Wang Q., Chen T., Du H., Yan S., Qi B. (2026). Robust and Conductive Soybean Protein Isolate-Dialdehyde Sodium Alginate Schiff Base Hydrogel Enhanced by the Hofmeister Effect for Flexible Sensors. Chem. Eng. J..

[146] Ma Q., Wang K., Hazra R.S., Chen Y., Wang Y., Jiang L. (2026). Soy Protein Derived Double Network (Dn) Ionic Conductive Organohydrogel toward Flexible Strain Sensor. Int. J. Biol. Macromol..

[147] Hu M., Deng Y., Qian X., Ye D., Jiang X., Xiao G. (2024). One-Pot Preparation of Strong, Tough, Frost-Resistant and Recyclable Organohydrogels Via Hofmeister Effect and Its Application for Electronic Devices. Eur. Polym. J..

[148] Li H., Fan R., Zhang F., Cui Z., Li J., Kang L., Li J., Huang Q., Tian D. (2024). Soybean Protein-Reinforced Highly Extensible and Self-Adhesive Hydrogels with Ultra-Sensitive Strain Sensing for Flexible Electronic Devices. Ind. Crops Prod..

[149] Wang Q., Chen T., Li Y., Du H., Li J., Yan S., Qi B. (2026). Fully Biobased Antimicrobial Ionic Conductive Hydrogel: Achieving Tunable Mechanical and Conductive Properties Via a Double-Network Structure Strategy for Flexible Sensors. Chem. Eng. J..

[150] Li J., Zhang J., Yan Y., Li A., Ren L. (2022). Fabrication of Heteroatom-Self-Doped Hierarchical Porous Carbon from Soy Protein Isolate Hydrogel for High-Performance Supercapacitors Via a Double-Effect Strategy of Template-Activation. Microporous Mesoporous Mater..

[151] Wang J., Xun Z., Zhao C., Liu Y., Gu J., Huo P. (2022). Converting Soy Protein Isolate into Biomass-Based Polymer Electrolyte by Grafting Modification for High-Performance Supercapacitors. Int. J. Biol. Macromol..

[152] Duan Y., Long J., Li Y., Tian X., Li J., Fang Z., Wang J., Huo P. (2024). Lignin/Soy Protein Isolate-Based Hydrogel Polymer Electrolytes for Flexible Solid-State Supercapacitors with Low Temperature Resistance. J. Solid State Electrochem..

[153] Dorishetty P., Balu R., Gelmi A., Mata J.P., Dutta N.K., Choudhury N.R. (2021). 3d Printable Soy/Silk Hybrid Hydrogels for Tissue Engineering Applications. Biomacromolecules.

[154] Ma Q., Wang K., Mohawk D., Mahoney A., Chen Y., Jiang L. (2024). Development of Dual-Curable Cellulose Nanofibrils-Reinforced Soy Protein Resins for 3d Printing. Ind. Crops Prod..

[155] Arain M.M., Khan A., Zahoor Z., Huang S., Chen Q., Rao D., Mei X., Pan S., Liu F. (2026). Effect of Pectin on Emulsifying Properties of Soy Protein Isolate: Tailoring Emulsion Gel Inks for 3d Printed Food Systems. Food Hydrocoll..

[156] Xu Y., Zhao J., Zhang M., Sun Y., Li X., Gao K., Liang J. (2025). *Naematelia aurantialba* Polysaccharide: Enhancing 3d Printability of Soy Protein Gel Via Rheology and Interactions Improvement. Int. J. Biol. Macromol..

[157] Barekat S., Ubeyitogullari A. (2025). Developing Hydrophobic-Hydrophilic Protein Structures by 3d Food Printing of Sorghum and Soy Protein Gels. J. Food Eng..

[158] Wang J., Zhang L., Tang H., Liu Y., Wang Y., Wang X., Gao M. (2025). Novel Biodegradable Extinguishing Gel: Preparation, Fire-Extinguishing Performance and Mechanism Study. Colloids Surf. A.

[159] Zhang H., Zhang T., Zhang X., Xu J., Peng X., Guo S. (2025). Powder-Level Mechanisms of Soy Protein Isolate Functional Deterioration During Storage: The Interplay of Aggregation and Oxidative Stress. Food Chem..

[160] Duque-Estrada P., Kyriakopoulou K., de Groot W., van der Goot A.J., Berton-Carabin C.C. (2020). Oxidative Stability of Soy Proteins: From Ground Soybeans to Structured Products. Food Chem..

[161] Zheng D., Jennifer R., Yiping T., Benu S., Xudong C. (2016). Sterilization Techniques for Biodegradable Scaffolds in Tissue Engineering Applications. J. Tissue Eng..

